# Targeting Histamine H4 Receptor in the Rostral Ventromedial Medulla to Relieve Hypertension

**DOI:** 10.1002/advs.202508176

**Published:** 2025-09-29

**Authors:** Ying Shi, Yang‐Xun Zhang, Jun‐Yi Chen, Sai Ma, Wei‐Xuan Xue, Wei Li, Qian‐Xiao Li, Bo Song, Ya‐Ting Li, Hong‐Yu Ma, Shu‐Tao Xie, Hong‐Zhao Li, Di‐Jun Chen, Qi‐Peng Zhang, Hui‐Jie Ma, Jian‐Jun Wang, Lei Yu, Xiao‐Yang Zhang, Jing‐Ning Zhu

**Affiliations:** ^1^ State Key Laboratory of Pharmaceutical Biotechnology National Resource Center for Mutant Mice Department of Anesthesiology, Nanjing Drum Tower Hospital Institute for Brain Sciences and Department of Physiology, School of Life Sciences Nanjing University 163 Xianlin Avenue Nanjing 210023 China; ^2^ Institute of Physical Education Jiangsu Second Normal University 6 Xinhe West Road Nanjing 211200 China; ^3^ Chemistry and Biomedicine Innovation Center (ChemBIC) ChemBioMed Interdisciplinary Research Center Nanjing University 163 Xianlin Avenue Nanjing 210023 China; ^4^ Department of Physiology Hebei Medical University 361 Zhongshan East Road Shijiazhuang 050017 China

**Keywords:** histamine, histamine H4 receptor, hypertension, rostral ventromedial medulla, stress

## Abstract

Refractory hypertension often involves centrally driven sympathetic augmentation. Yet effective central targets for the treatment of hypertension are still scarce. Here, the role of the histamine H4 receptor (H4R) is explored, the newest member of the histamine receptor family, in central cardiovascular regulation. Analysis of single‐nucleus RNA‐sequencing datasets of human brains and RNAscope assays of rat brains reveal a conservative expression of H4R in the rostral ventromedial medulla (RVMM), a key sympathetic cardiovascular center. Optoactivation of RVMM histaminergic afferents evokes depressor and bradycardic responses via H4R, whose activation excites GABAergic presympathetic neurons by transient receptor potential vanilloid 1 (TRPV1). Intranasal delivery of H4R agonist produces sustained blood pressure‐lowering effects in free‐moving spontaneously hypertensive rats and stress‐induced hypertensive rats. The findings reveal an antihypertensive role of RVMM histamine H4R and a potential central therapeutic target for hypertension.

## Graphical abstract

H4R and its downstream TRPV1 channel mediate the excitatory effect of TMN histaminergic afferent inputs on GABAergic sympathoinhibitory neurons in RVMM. Intranasal delivery of selective H4R agonist VUF 8430 suppresses renal sympathetic outflow and elicits sustained depressor and bradycardia responses in SHRs and CUMS‐induced hypertensive rats.

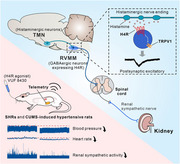

## 1 Introduction

Hypertension has long been recognized as a high risk for cardiovascular diseases and a major health burden worldwide.^[^ ^1^ ^]^ Despite recent advances in antihypertensive therapies, more than half of the treated patients failed to achieve adequate blood pressure control.^[^ ^2^ ^]^ Compared with subjects with normotension or non‐resistant hypertension, patients with refractory hypertension have been reported to be characterized by increased sympathetic nerve activity,^[^ ^3^, ^4^, ^5^ ^]^ highlighting the potential of targeting central mechanisms underlying sympathetic overactivity as a therapeutic strategy. It has been well known that both tonic sympathetic activation and arterial pressure control critically depend on the vasomotor centers in the brainstem.^[^ ^6^ ^]^ Structural and functional alterations in the brainstem have been observed in hypertensive patients,^[^ ^7^ ^]^ and clinical cases of neurogenic hypertension following brainstem injury further support the central contribution to blood pressure dysregulation.^[^ ^8^ ^]^ However, centrally acting antihypertensive medications with few side effects are still lacking, hindering effective treatment of hypertensive conditions driven by sympathetic drive.

Evidence suggests that the central histaminergic system may contribute to cardiovascular regulation. Neuroanatomically, histamine‐containing neurons, originating exclusively from the tuberomammillary nucleus (TMN) of the hypothalamus, send direct axonal projections to the major vasomotor and sympathetic premotor centers in the brainstem, specifically the rostral ventrolateral medulla (RVLM) and rostral ventromedial medulla (RVMM).^[^ ^9^, ^10^, ^11^ ^]^ Moreover, increasing central histamine levels, either via oral administration or directly intraventricular injection of l‐histidine, a histamine precursor, significantly reduced mean arterial pressure (MAP) in spontaneously hypertensive rats (SHRs).^[^ ^12^ ^]^ As is well known, histamine acts through four different G‐protein‐coupled receptors, namely H1R, H2R, H3R, and H4R, constituting a most outstanding receptor family for drug discovery.^[^ ^13^ ^]^ Among the four subtypes of histamine receptors, H1R, H2R, and H3R have been well recognized for their key roles in the regulation of various brain functions, including sleep‐wake cycle, reward, and motivation, emotion, metabolism, as well as motor control.^[^ ^14^, ^15^, ^16^, ^17^ ^]^ Accordingly, ligands binding at H1R, H2R, and H3R have already been widely developed and used in the clinical treatment of allergic, peptic ulcer, and vestibular disorders, respectively.^[^ ^18^, ^19^, ^20^, ^21^ ^]^ Furthermore, as a newly identified member of histamine receptors at the turn of the millennium, H4R has received increasing attention.^[^ ^22^, ^23^ ^]^ Distinct from the broad central and peripheral distributions and diverse functions of the other three histamine receptor subtypes, H4R is found mainly in peripheral immune cells and blood‐forming tissues to mediate chronic inflammation.^[^ ^24^, ^25^, ^26^ ^]^ More importantly, the central distribution of H4R appears to be very few and highly restricted,^[^ ^27^, ^28^ ^]^ which may indicate a specialized central role of the receptor. Notably, the histamine‐induced decrease in MAP has been observed to be reversed by intracerebroventricular injection of thioperamide,^[^ ^12^ ^]^ which was reported first to be a selective antagonist for H3R and identified later as an antihistamine at H3R and H4R with similar potential.^[^ ^29^ ^]^ We thus propose a possible role of central H4R in blood pressure control, which may provide a highly specialized central target for hypertension.

In this study, we employed histidine decarboxylase (HDC)‐Cre transgenic rats, *Hrh4*‐knockout (KO) mice, and two hypertensive models (SHRs and stress‐induced hypertensive rats) to investigate H4R as a potential central target for hypertension management. By integrating analyses of publicly available single‐nucleus RNA sequencing (snRNA‐seq) datasets, RNA‐protein co‐detection assays, electrophysiology, optogenetics, intranasal drug delivery, and telemetric cardiovascular monitoring, we identified conserved H4R expression in the medullary cardiovascular center across species and elucidated its functional role in blood pressure regulation. Activation of H4R in the RVMM elicited depressor and bradycardic responses in normotensive rats and mitigated hypertension in both models by exciting RVMM neurons through transient receptor potential vanilloid 1 (TRPV1)‐mediated pathways, thereby reducing renal sympathetic nerve activity (RSNA). These findings uncover a previously unrecognized central role of H4R in cardiovascular control and highlight its potential as a therapeutic target for hypertension.

## 2 Results

### 2.1 Histamine H4R on RVMM Neurons Mediates Depressor and Bradycardia responses

**Figure 1:**
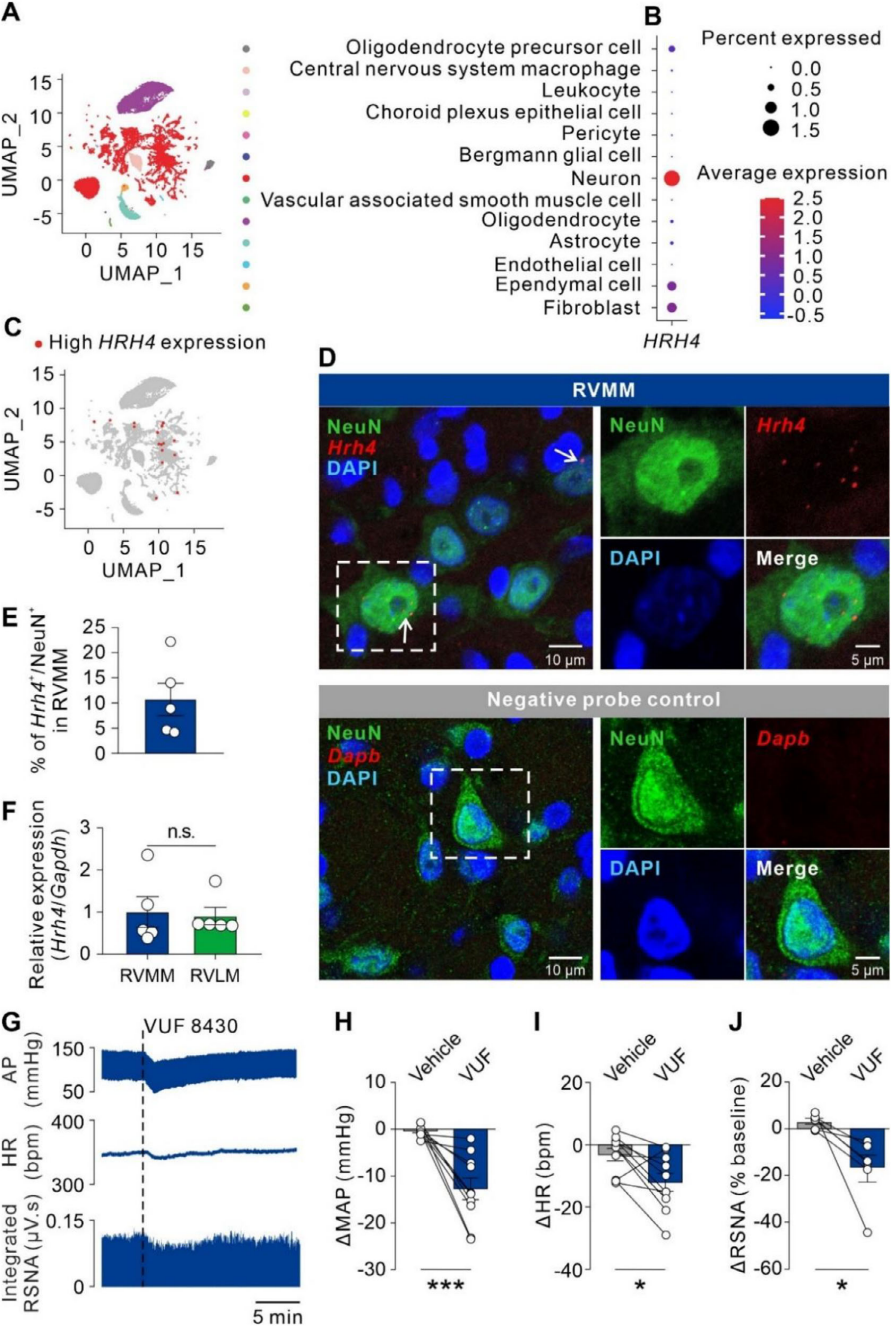
H4R is expressed in the RVMM and induces a decrease of MAP, HR, and RSNA in normal rats in vivo. A) Major cell types identified from a single nucleus RNA sequencing dataset are shown as clusters in a 2D UMAP embedding. B) The dot plot showed H4R expression levels in each cell cluster. C) UMAP plots showed a high H4R expression level in neurons. D) RNA‐protein integrated co‐detection showing H4R mRNA localized on RVMM (upper panel). Arrows showed neurons expressing H4R mRNA. A negative control probe targeting dihydrodipicolinate reductase (*Dapb*) gene was used to ensure that there was no background staining related to the RNAscope assay (lower panel). Scale bar, 10 µm (overview) and 5 µm (magnified inset). E) Percentage of neurons expressing *Hrh4* in the RVMM (*n* = 5). F) Relative expression of *Hrh4* in RVMM and RVLM (*n* = 5, *P* > 0.05, two‐tailed unpaired student's t‐test). G) Raw traces illustrating changes in AP, HR, and RSNA induced by unilateral microinjection of VUF 8430 into RVMM. H–J) Group data of maximal responses of MAP (H; ***n*** = 10, *P* = 0.0009), HR (I; *n* = 10, *P* = 0.0153), and RSNA (J; *n* = 6, *P* = 0.0272) after vehicle or VUF 8430 microinjection into RVMM (two‐tailed paired student's t‐test). Group data were presented as means ± S.E.M. *P < 0.05, ***P < 0.001, n.s. no significance.

We first analyzed the expression of H4R in the human medulla oblongata (myelencephalon), which includes the cardiac and vasomotor centers regulating heart rate and blood pressure, by using a snRNA‐seq dataset from Human Brain Cell Atlas v1.0.^[^ ^30^ ^]^ A total of 203472 cells were clustered into 13 unique groups by Louvain clustering (scikit‐network) (**Figure** **1**A), and a relatively higher expression of the H4R gene (*HRH4*) was observed within the neuron cluster (Figure 1B,C).

We further employed RNAscope combined with immunofluorescence to determine whether H4R is distributed in the neurons of RVMM and RVLM, two major sympathetic cardiovascular centers in the medulla oblongata,^[^ ^6^ ^]^ in rats. As shown in Figure 1D and Figure S1A (Supporting Information), *Hrh4* probe fluorescence signals were detected in both RVMM and RVLM, and co‐localized with the NeuN‐positive neurons at the percentage of 10.79% ± 3.36% in RVMM (Figure 1E) and 8.53% ± 1.62% in RVLM (Figure S1B, Supporting Information), while a negative control probe was used to ensure no background staining related to the RNAscope assay (Figure 1D). We quantified the relative expression of *Hrh4* by RT‐qPCR, and found a comparable expression of *Hrh4* in the RVMM and RVLM (Figure 1F). These rat data, together with the transcriptome analysis of human medulla oblongata, suggest that the expression of H4R in the medulla oblongata may be conserved across mammalian species.

Next, we assessed whether histamine H4R in RVMM and RVLM contributes to cardiovascular regulation. Microinjection of VUF 8430, a selective histamine H4 receptor agonist, into the RVMM elicited a remarkable decrease in MAP compared with injection of vehicle that did not induce any change in MAP (Vehicle vs VUF: −0.37 ± 0.37 mmHg vs −12.72 ± 2.32 mmHg; Figure 1G,H). Moreover, a significant reduction in average peak HR was observed after microinjection of VUF 8430 into RVMM, compared with vehicle injection (Vehicle vs VUF: −3.11 ± 2.00 bpm vs −12.01 ± 2.89 bpm; Figure 1G,I). We also found that intra‐RVMM microinjection of VUF 8430 induced a significant decrease in RSNA, compared with vehicle (Vehicle vs VUF: 3.17% ± 1.24% vs −17.10% ± 5.78%; Figure 1G,J), indicating a suppression of central sympathetic outflow by activation of H4R in RVMM. However, microinjecting VUF 8430 into the RVLM did not influence MAP, HR and RSNA (Vehicle vs VUF: −0.04 ± 0.86 mmHg vs 0.29 ± 1.03 mmHg; 2.79 ± 1.90 bpm vs −1.62 ± 2.14 bpm; 1.33% ± 1.76% vs −0.23% ± 1.77%; Figure S2, Supporting Information). These data indicate that activation of histamine H4R in RVMM rather than RVLM induces depressor and bradycardia responses.

In addition, we evaluated whether the other histamine receptor subtypes, i.e., H1R, H2R, and H3R, were involved in cardiovascular control mediated by RVMM. Intra‐RVMM microinjection of 2‐PyEA, dimaprit, or RAMH (selective agonists for H1R, H2R, and H3R, respectively), had no impact on MAP and HR (Vehicle vs 2‐PyEA: 0.82 ± 0.78 mmHg vs 0.26 ± 0.80 mmHg, 0.36 ± 2.70 bpm vs −1.82 ± 1.75 bpm; Vehicle vs dimaprit: 0.89 ± 0.88 mmHg vs 0.02 ± 0.86 mmHg, 1.14 ± 2.87 bpm vs 0.23 ± 2.56 bpm; Vehicle vs RAMH: 0.77 ± 0.86 mmHg vs −0.84 ± 0.56 mmHg, 0.18 ± 2.48 bpm vs −3.04 ± 1.74 bpm; Figure S3, Supporting Information). These results further suggest that H4R, but not H1R, H2R, and H3R, contributes to the cardiovascular regulation of RVMM.

### 2.2 Optogenetic Activation of TMN‐RVMM Histaminergic Afferents Produces Depressor and Bradycardia Responses via H4R

**Figure 2:**
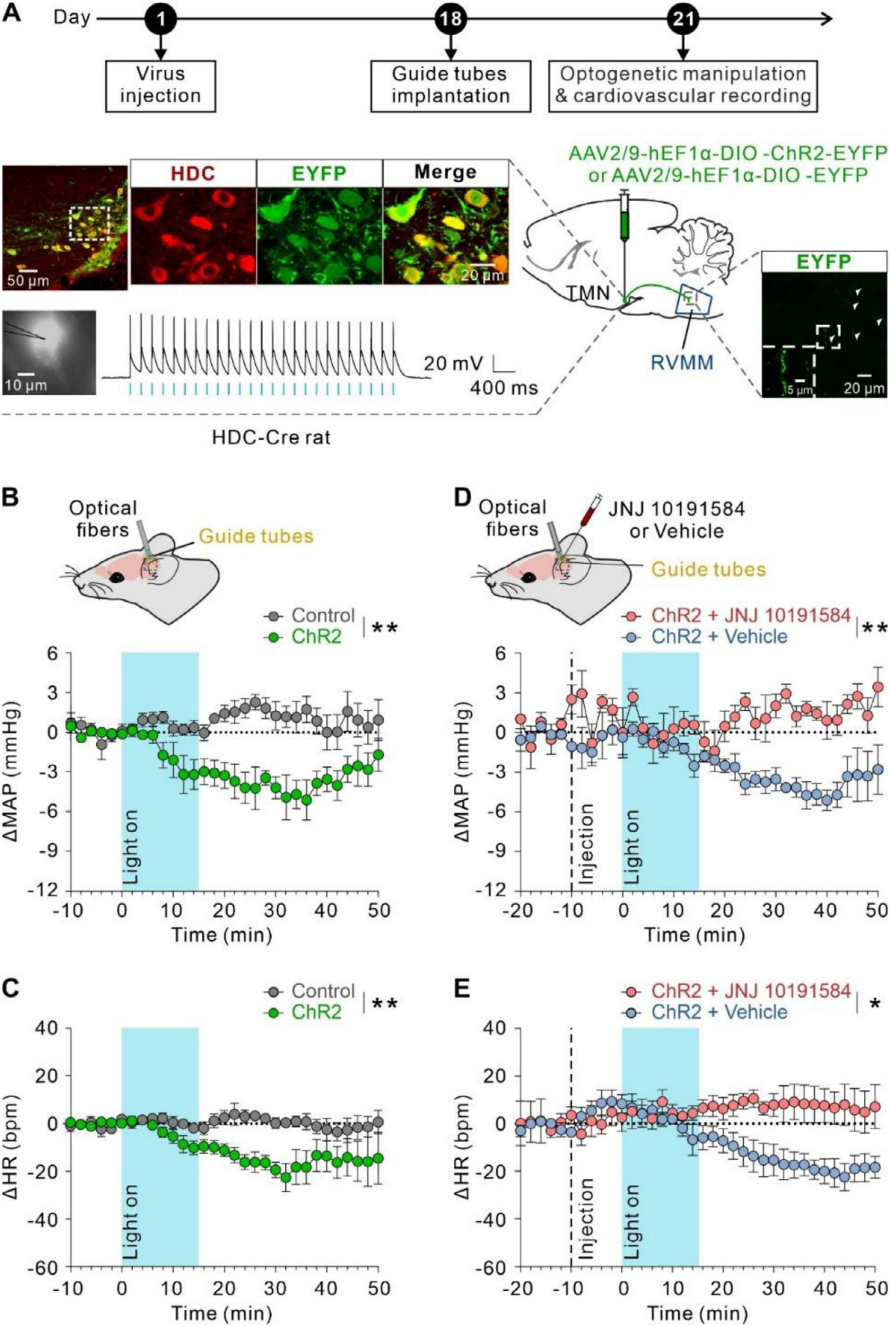
TMN‐RVMM histaminergic circuitry mediates depressor and bradycardia response via H4R. A) Scheme of the experimental paradigm showing optogenetic and pharmacology manipulation with cardiovascular recording in HDC‐Cre rats in vivo. In the lower left panel, immunofluorescence staining showed the specific expression of Cre recombinase‐dependent virus ChR2‐ EYFP in HDC‐positive neurons; scale bar, 50 µm (left) and 20 µm (right). And optogenetic stimulation with 5 Hz blue light pulses consistently elicited precisely time‐locked action potentials in ChR2‐expressing histaminergic neurons within the TMN; scale bar, 10 µm. In the lower right panel, fluorescence EYFP labeled histaminergic afferent fibers distributed in RVMM; scale bar, 20 µm (overview) and 5 µm (magnified inset). B and C) Blue light stimulation (blue shadow) reduced MAP (B; *n* = 6, *P* = 0.0033) and HR (C; *n* = 6, *P* = 0.0049) in the ChR2 group (green circles), rather than the control group (gray circles). D and E) Microinjection of selective H4R antagonist JNJ 10191584 (red circles), instead of vehicle (0.02% DMSO in ACSF, blue circles), into RVMM significantly blocked the optogenetic activation‐induced depressor (D; *n* = 5, *P* = 0.0035) and bradycardia response (E; *n* = 5, *P* = 0.0210). Group data were presented as means ± S.E.M and analyzed by repeated measures two‐way ANOVA with Bonferroni's multiple comparisons test. *P < 0.05, **P < 0.01.

To determine the role of histaminergic afferent inputs into RVMM in cardiovascular regulation, we generated a transgenic rat line expressing Cre recombinase under the control of the HDC promoter, the key enzyme for endogenous histamine synthesis, as described previously.^[^ ^15^ ^]^ Hypothalamic TMN histaminergic neurons in HDC‐Cre rats were then selectively transduced with a ChR2‐EYFP virus to examine the effects of optogenetic activation of TMN‐RVMM histaminergic projections on cardiovascular activity (**Figure** **2**A). ChR2‐EYFP and control virus were found to be selectively expressed in the hypothalamic TMN and colocalized with HDC‐positive neurons in HDC‐Cre rats (Figure 2A). Whole‐cell patch‐clamp recordings further confirmed the functional efficacy of optogenetic stimulation, showing high‐fidelity action potential firing in response to 5 Hz blue light pulses (10 ms pulses) (Figure 2A), though fidelity progressively declined at higher frequencies (Figure S4, Supporting Information). Furthermore, histaminergic afferents, labeled with fluorescence EYFP, possessed prominent varicosities in RVMM (Figure 2A). Notably, optogenetic activation (5 Hz and 10 Hz) of TMN‐RVMM histaminergic afferent terminals induced a significant fall in MAP (Figure 2B; Figure S5, Supporting Information) and HR (Figure 2C; Figure S5, Supporting Information), compared with ChR2‐negative control rats. It suggests that TMN histaminergic neurons send direct projections to RVMM to produce depressor and bradycardia responses. We further evaluated the contribution of H4R to the cardiovascular effects elicited by optogenetic activation of TMN‐RVMM histaminergic projections. Microinjection of the selective H4R antagonist JNJ 10191584 into RVMM nearly totally abolished the depressor (Figure 2D) and bradycardia (Figure 2E) responses produced by optogenetic manipulation. These results suggest that TMN‐RVMM histaminergic projections may hold a key position in the endogenously negative regulation of cardiovascular activity via the activation of histamine H4R.

### 2.3 Activation of H4R Evokes a Postsynaptic Excitatory Response of Neurons in RVMM

**Figure 3:**
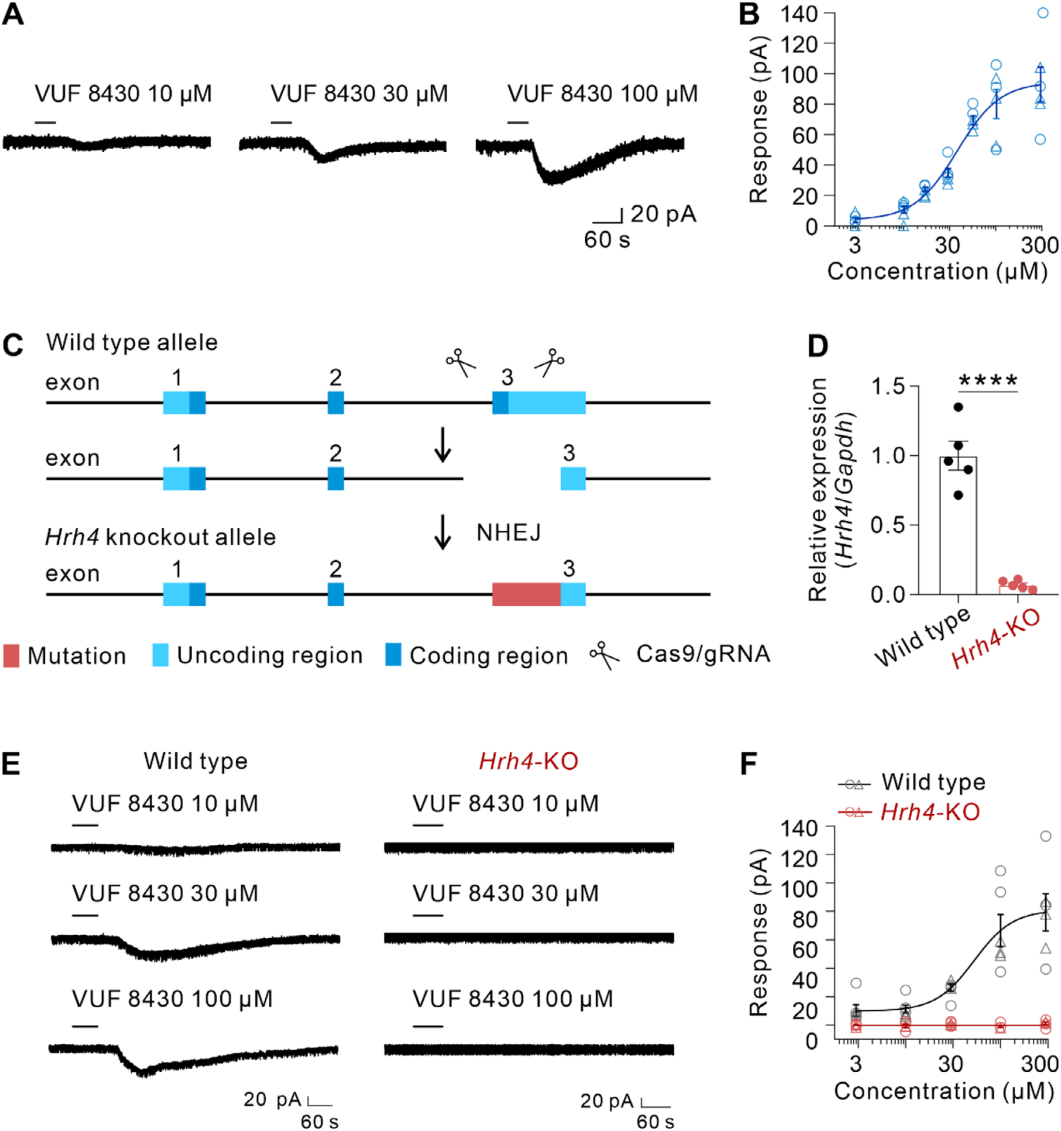
The selective H4R agonist VUF 8430 evoked inward currents in a concentration‐dependent manner on RVMM neurons. A) Infusion of H4R agonist VUF 8430 evoked inward currents on an RVMM neuron. B) Concentration‐response curves of VUF 8430 in recorded RVMM neurons (*n* = 6; circles for 3 male rats and triangles for 3 female rats). C) Scheme of *Hrh4*‐KO mice strain. NHEJ, non‐homologous end joining. D) Relative expression of brain *Hrh4* in wildtype mouse and *Hrh4*‐KO mouse (*n* = 5 for per group, *P* < 0.0001; two‐tailed unpaired student's t‐test). E) Raw tracing showed inward current on RVMM neurons induced by perfusion of 10, 30, and 100 µm VUF 8430 in wild‐type and *Hrh4*‐KO mouse, respectively. F) Application of VUF 8430 evoked concentration‐dependent inward current on RVMM neurons in wildtype mouse (*n* = 6, gray circles for 3 male rats and gray triangles for 3 female rats) while had no effects on *Hrh4*‐KO mouse (*n* = 5, red circles for 3 male rats and red triangles for 2 female rats). Group data were presented as means ± S.E.M, ****P < 0.0001.

To determine the cellular mechanism underlying the histamine H4R‐mediated cardiovascular effects, we employed whole‐cell patch clamp recording to examine the impact of H4R activation on the electrophysiological activities of RVMM neurons. As shown in **Figure** **3**A, the selective histamine H4R agonist VUF 8430 excited RVMM neurons (28/33) in a concentration‐dependent manner (Figure 3A; Figure S6A, Supporting Information). Application of 10 µM, 30 µM, and 100 µM VUF 8430 elicited an inward current of 10.60 ± 2.41 pA, 34.65 ± 2.96 pA, and 80.12 ± 9.60 pA, respectively (Figure 3A,B). Fitting the concentration‐response curves from RVMM neurons provided EC_50_ value for VUF 8430 of 37.63 µM (Figure 3B). To further confirm the pharmacological specificity of VUF 8430, we conducted parallel experiments in *Hrh4*‐KO mice, which exhibited complete ablation of *Hrh4* expression (Figure 3C,D). While VUF 8430 elicited concentration‐dependent responses in wildtype mice (EC_50_ = 52.81 µM), comparable to the rat data, it failed to activate RVMM neurons in KO mice even at 300 µM (Figure 3E,F). These results provide definitive evidence that VUF 8430‐induced excitatory effects in RVMM neurons are specifically mediated through H4R, rather than through other histamine receptor subtypes or nonspecific mechanisms. On the other hand, in line with the result that RVLM H4R activation did not cause any cardiovascular response, VUF 8430 (up to 300 µM) had no significant effect on RVLM neurons (Figure S6B, Supporting Information), which may account for why activation of H4R in RVLM does not influence cardiovascular activity. These results suggest that activation of histamine H4R may excite neurons in the RVMM instead of RVLM.

**Figure 4:**
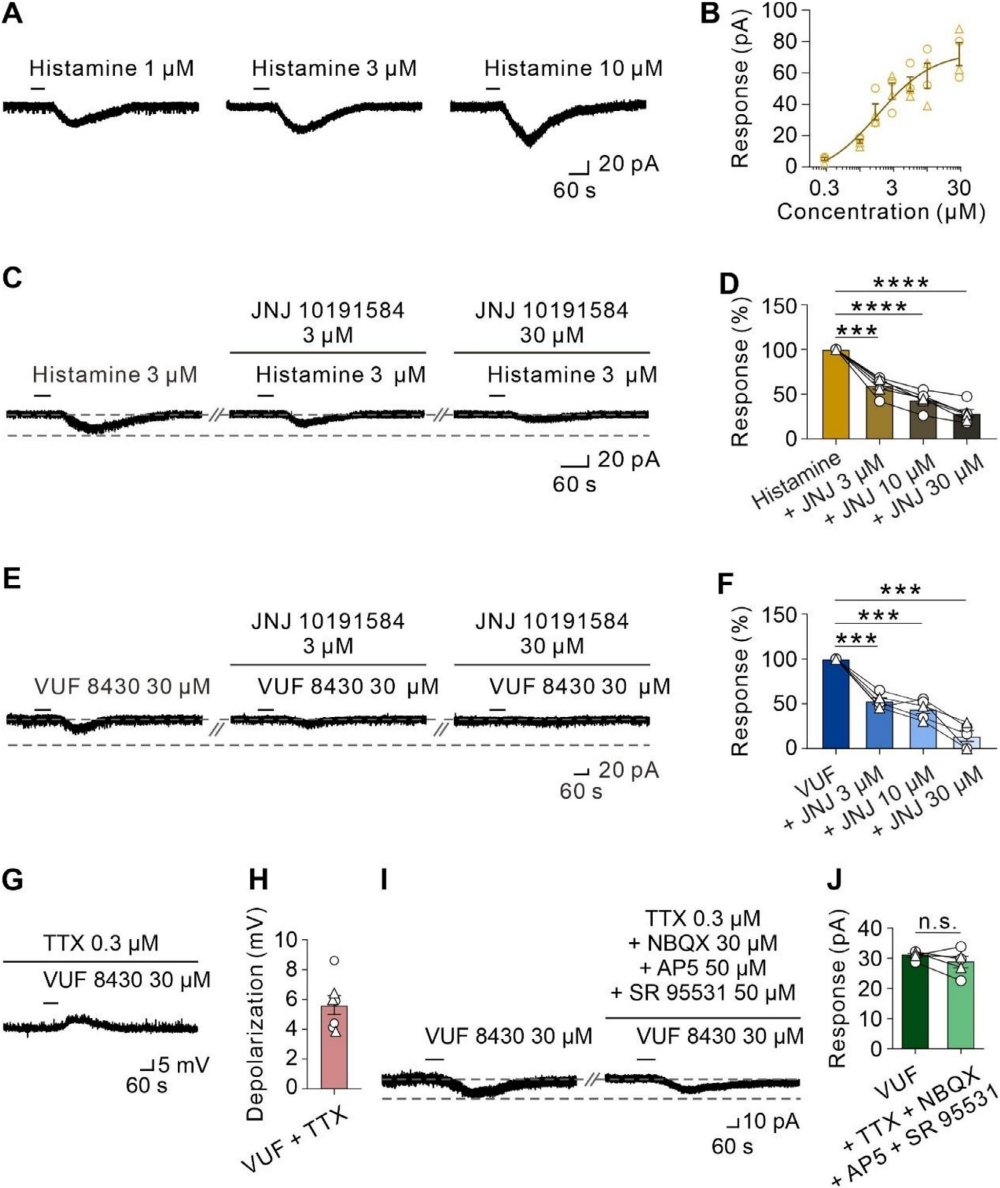
The selective H4R antagonist JNJ 10191584 blocks the inward currents evoked by histamine and VUF 8430. A) Raw tracing of inward currents evoked by histamine infusion in an RVMM neuron. B) Concentration‐response curves of histamine in 4 recorded RVMM neurons (circles for 2 male rats and triangles for 2 female rats). C and D) Raw traces and group data showed that JNJ 10191584 blocked the histamine‐induced inward currents in a dose‐dependent manner on an RVMM neuron (JNJ 3 µM: *n* = 6, *P* = 0.0005; JNJ 10 µM: *n* = 6, *P* < 0.0001; JNJ 30 µM: *n* = 6, *P* < 0.0001; circles for 3 male rats and triangles for 3 female rats; one‐way ANOVA with Bonferroni's multiple comparisons test). E and F) Raw traces and group data showed that JNJ 10191584 blocked the VUF 8430‐evoked inward currents in a dose‐dependent manner on an RVMM neuron (JNJ 3 µM: *n* = 5, *P* = 0.0006; JNJ 10 µM: *n* = 5, *P* = 0.0010; JNJ 30 µM: *n* = 5, *P* = 0.0004; circles for 3 male rats and triangles for 2 female rats; one‐way ANOVA with Bonferroni's multiple comparisons test). G and H) Raw traces and group data showed that VUF 8430 induced a strong depolarization in the presence of TTX under the current clamp mode in an RVMM neuron (*n* = 7; circles for 4 male rats and triangles for 3 female rats). I and J) Bath with TTX, NBQX, AP5, and SR 95531 did not affect VUF 8430‐induced inward current in an RVMM neuron. Group data in (J) (*n* = 5, *P* = 0.2406; circles for 3 male rats and triangles for 2 female rats; two‐tailed paired student's t‐test). Data were presented as means ± S.E.M. ***P < 0.001, ****P < 0.0001, n.s. no significance.

We further explored the contribution of H4R to the effect of histamine on RVMM neurons by application of JNJ 10191584, a highly selective histamine H4R antagonist. As shown in **Figure** **4**A, histamine concentration‐dependently elicited an inward current in RVMM neurons (30/44) with an EC50 of 1.80 µM (Figure 4A,B; Figure S6A, Supporting Information). JNJ 10191584 produced a concentration‐dependent inhibition on the excitatory response of RVMM neurons elicited by 3 µM histamine, which was close to the EC_50_ for histamine (Figure 4C). Perfusing the slice with 3 µM, 10 µM, and 30 µM JNJ 10191584 significantly reduced the histamine‐induced inward current on RVMM neurons to 59.77% ± 3.96%, 43.70% ± 3.92% and 28.57% ± 4.22%, respectively (Figure 4D). It thus suggests that H4R is actively involved in the excitatory effect of histamine on RVMM neurons. Moreover, consistent with the results of blocking the effect of histamine, JNJ 10191584 (3, 10, and 30 µm) also blocked the inward current induced by 30 µM VUF 8430 in a concentration‐dependent manner, with a reduction of 53.07% ± 3.60%, 44.00% ± 4.88% and 13.95% ± 6.06%, respectively (Figure 4E,F), further confirming the excitatory effect on RVMM neurons mediated by the histamine H4R activation.

Considering that histamine H4R is a postsynaptic receptor,^[^ ^31^ ^]^ we next determined whether the H4R‐induced excitation on RVMM neurons is a direct postsynaptic effect. In the presence of 0.3 µM TTX under the current clamp mode, 30 µM VUF 8430 still induced a strong depolarization of 5.62 ± 0.63 mV in RVMM neurons (Figure 4G,H). Furthermore, we applied TTX (a selective voltage‐sensitive sodium channel blocker, 0.3 µM), together with SR 95531 (a selective GABA_A_ receptor antagonist, 50 µM), NBQX (a selective AMPA receptor antagonist, 30 µM) and AP5 (a selective NMDA receptor antagonist, 50 µM), to block synaptic transmission in voltage clamp mode, and observed that the VUF 8430‐induced inward current was not affected (Figure 4I,J). All these results indicate that activation of histamine H4R induces a direct postsynaptic excitatory effect on RVMM neurons.

### 2.4 H4R Activation Excites RVMM Neurons to Produce a Depressor and Bradycardia Response via TRPV1

**Figure 5:**
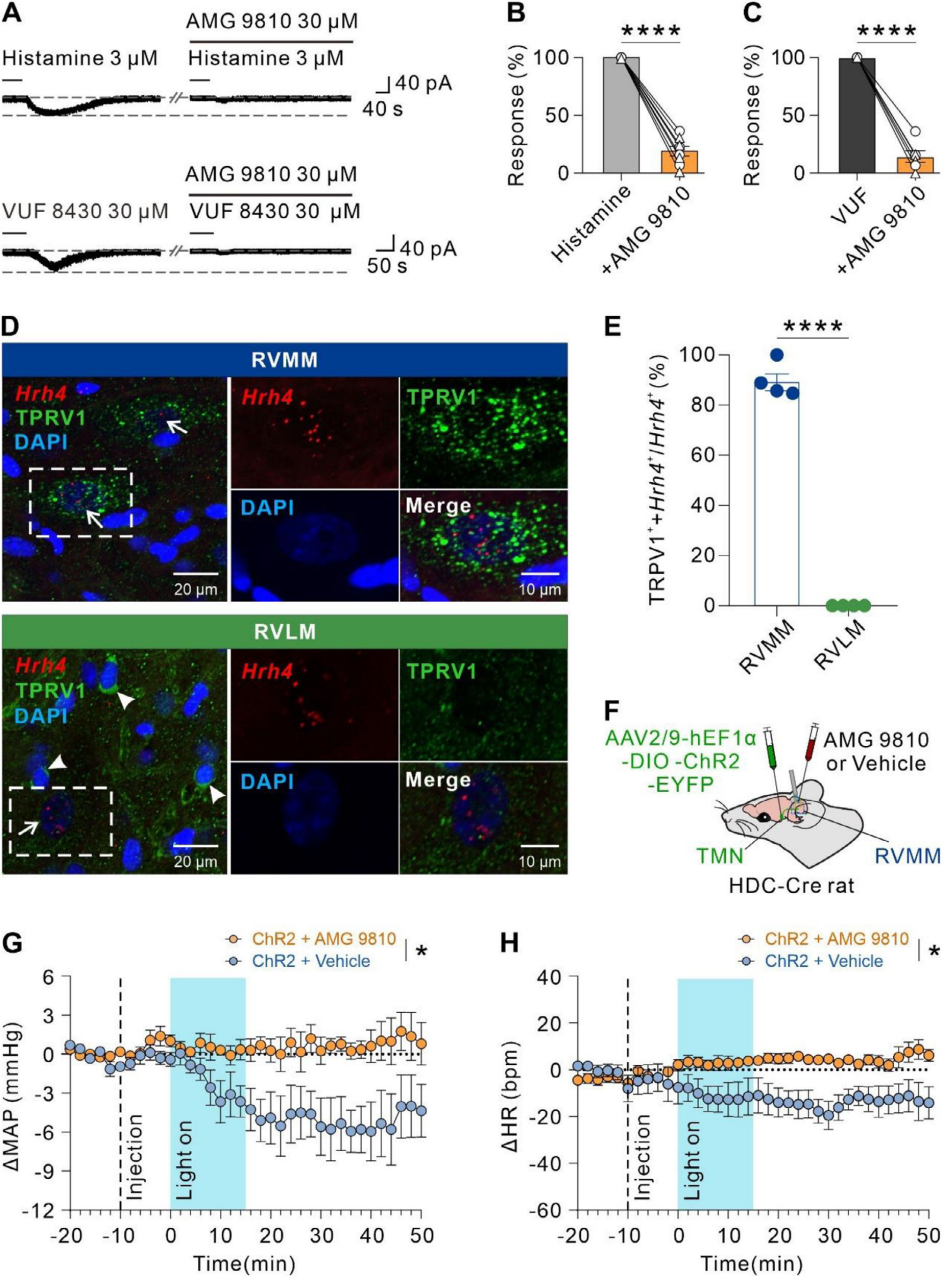
H4R activation excites RVMM neurons via the opening of TRPV1 channels. A) Raw tracing showed a blockage of histamine‐induced inward current and VUF‐induced inward current on RVMM neurons by a selective TRPV1 antagonist AMG 9810. B and C) Group data for (A) (B: *n* = 8, circles for 4 male rats and triangles for 4 female rats; *P* < 0.0001; C: *n* = 6, circles for 3 male rats and triangles for 3 female rats; *P* < 0.0001; two‐tailed paired student's t‐test). D) In situ hybridization combined with immunofluorescence images showed that *Hrh4* and TRPV1 proteins were co‐localized in RVMM rather than RVLM. Arrows showed neurons with fluorescent *Hrh4* probe signals. Arrowheads showed RVLM neurons immunoreactive for TRPV1 without *Hrh4* hybridization signals. Scale bar, 20 µm (left, overview) and 10 µm (right, magnified inset). E) Percentage of TRPV1 immunoreactivity in *Hrh4*‐expressing RVMM and RVLM neurons (*n* = 4, *P* < 0.0001; two‐tailed unpaired student's t‐test). F) Scheme of the optogenetic experimental paradigm. G and H) Microinjection of selective TRPV1 antagonist AMG 9810 (yellow circles), instead of vehicle (0.3% DMSO in ACSF, blue circles), in RVMM significantly blocked the light stimulation‐induced depressor and bradycardia response (G: *n* = 5, *P* = 0.0238; H: *n* = 5, *P* = 0.0272; repeated measures two‐way ANOVA with Bonferroni's multiple comparisons test). Group data were presented as means ± S.E.M. *P < 0.05, ****P < 0.0001.

The central ionic mechanism underlying the excitatory effect mediated by H4R activation remains unknown. Considering TRPV1 is involved in the H4R‐evoked excitatory effect in peripheral dorsal root ganglion (DRG) neurons,^[^ ^32^ ^]^ we perfused the brain slices with artificial cerebrospinal fluid (ACSF) containing AMG 9810, a selective antagonist of TRPV1, to assess whether TRPV1 mediated the excitation of RVMM neurons induced by H4R activation. We found that AMG 9810 (30 µM) nearly totally blocked the inward current in RVMM neurons induced by histamine (3 µM) from 100% to 18.93% ± 4.19% (**Figure** **5**A,B), and completely blocked the inward current induced by VUF 8430 (30 µM) from 100% to 14.27% ± 5.02% (Figure 5A,C), indicating that histamine H4R activation excites RVMM neurons via the opening of TRPV1. Furthermore, we examined the distribution of TRPV1 in RVMM, and observed that the vast majority (89.83% ± 3.51%) of RVMM neurons with *Hrh4* probe fluorescence signals were TRPV1‐immunopositive (Figure 5D,E), indicating a co‐expression of TRPV1 and H4R in the same RVMM neurons. Although TRPV1 was also detected to be expressed in the RVLM, *Hrh4* probe fluorescence signals were not distributed in the TRPV1‐immunopositive positive neurons (Figure 5D,E). These results suggest that TRPV1 mediates the excitatory effect of H4R activation on RVMM neurons. Furthermore, we determined the contribution of TRPV1 to the cardiovascular effects induced by optogenetic activation of RVMM histaminergic afferent terminals (Figure 5F). Intra‐RVMM microinjection of selective TRPV1 antagonist AMG 9810 completely blocked the depressor (Figure 5G) and bradycardia (Figure 5H) responses induced by activation of TMN‐RVMM histaminergic projections. Therefore, H4R and its downstream TRPV1 channel mediate the excitation of RVMM neurons and subsequent depressor and bradycardia responses induced by RVMM histaminergic inputs.

### 2.5 H4R Activation Excites GABAergic Neurons in RVMM to Suppress Renal Sympathetic Outflow

**Figure 6:**
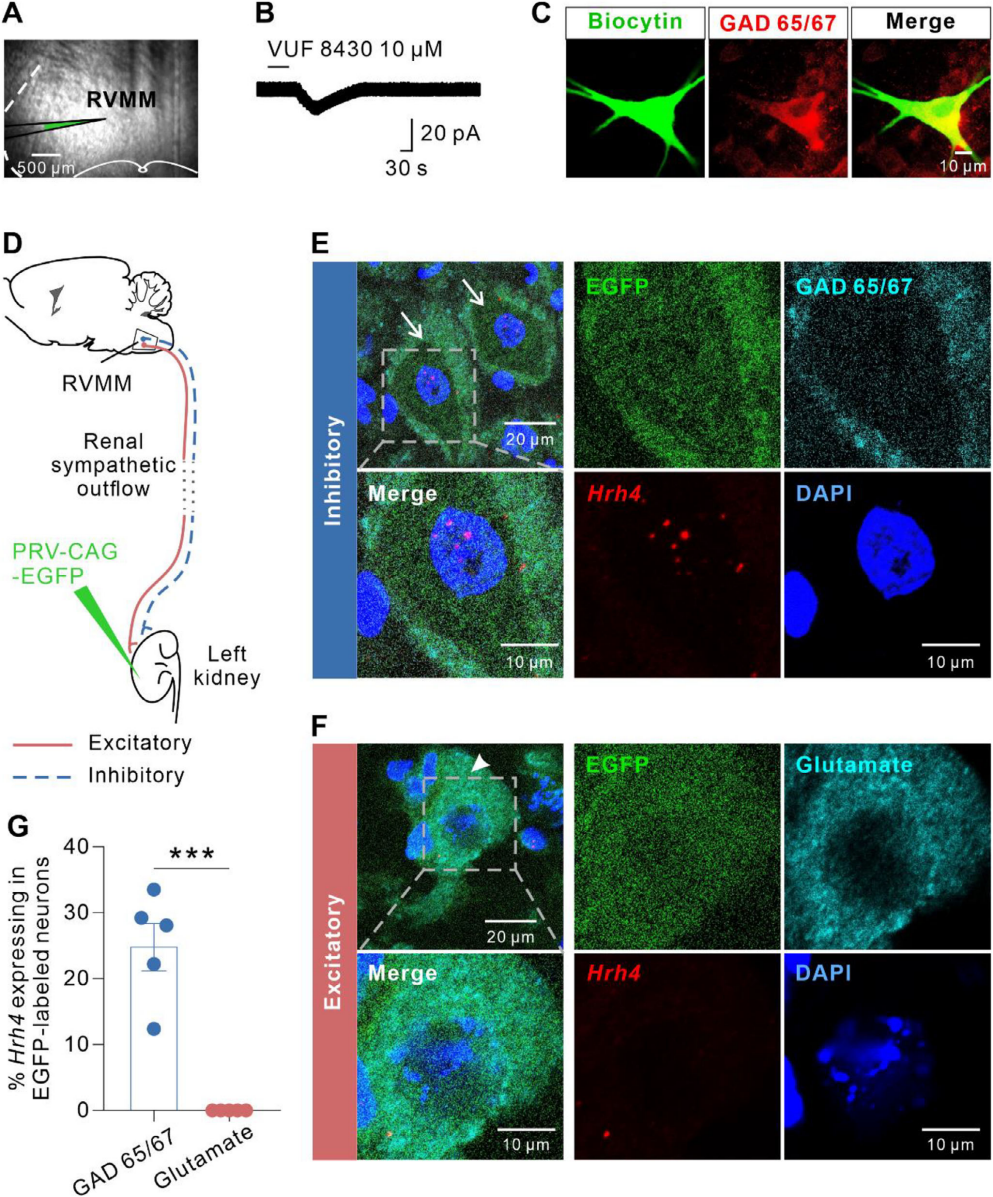
RVMM*^Hrh4^* neurons induce an inhibitory renal sympathetic outflow. A and B) RVMM neurons were recorded with borosilicate glass pipettes filled with an internal solution with 4% biocytin in the perfusing of 10 µM VUF 8430. Scale bar, 500 µm. C) Biocytin‐labeled RVMM neurons were GAD65/67 immunoreactive. Scale bar, 10 µm. D) Scheme of microinjection of retrograde transneuronal tracing virus, PRV‐CAG‐EGFP, into the left kidney for tracing sympathoinhibitory and sympathoexcitatory neurons in the RVMM. E and F) In situ hybridization combined with immunofluorescence images showed that EGFP‐labeled RVMM presympathetic neurons expressing *Hrh4* were GAD 65/67‐positive (E) rather than glutamate‐positive F). Arrows and arrowheads showed neurons with and without fluorescent *Hrh4* probe signals, respectively. Scale bar, 20 µm (overview) and 10 µm (magnified inset). G) Percentage of H4R mRNA expressing in PRV‐EGFP labeled RVMM neurons with GAD 65/67 immunoreactivity or glutamate immunoreactivity (*n* = 5, *P* = 0.0001; two‐tailed unpaired student's t‐test). Group data were presented as means ± S.E.M. ***P < 0.001.

Considering that depressor and bradycardia responses usually result from RSNA suppression,^[^ ^33^, ^34^ ^]^ we further determined the mechanism underlying the H4R‐mediated inhibition of sympathetic outflow. RVMM has been reported to contain excitatory and inhibitory presympathetic neurons.^[^ ^35^ ^]^ Therefore, we recorded the whole‐cell current of RVMM neurons with pipettes containing biocytin and observed that all recorded neurons excited by VUF 8430 perfusion were GAD65/67 immunoreactive (**Figure** **6**A–C), suggesting that activation of H4R excites GABAergic neurons in the RVMM. We next evaluated whether the RVMM GABAergic neurons expressing H4R were sympathoinhibitory neurons. We microinjected pseudorabies virus (PRV), a retrograde transsynaptic tracer, into the sympathetically innervated renal parenchyma to identify the glutamate‐positive sympathoexcitatory neurons and the GAD 65/67‐positive sympathoinhibitory neurons in RVMM (Figure 6D; Figure S7, Supporting Information). RNAscope in situ hybridization showed that *Hrh4* probe fluorescence signals were localized in retrogradely labeled RVMM GABAergic neurons (GAD 65/67‐positive), representing 25.09% ± 3.65% of this subpopulation (Figure 6E–G). In striking contrast, glutamatergic sympathoexcitatory neurons projecting to the kidney showed no detectable *Hrh4* mRNA expression (Figure 6F,G). These results suggest that H4R activation may directly excite RVMM GABAergic presympathetic neurons, which may in turn suppress renal sympathetic outflow and induce depressor and bradycardia responses.

### 2.6 Activation of H4R in RVMM Constantly Ameliorates Hypertension in Free‐Moving SHRs and CUMS‐Induced Hypertensive Rats

Finally, we employed SHR and chronic unpredictable mild stress (CUMS)‐induced hypertensive rats, two commonly used animal models of primary hypertension,^[^ ^36^, ^37^ ^]^ to explore the therapeutic potential of targeting RVMM H4R to treat hypertension. Exaggerated sympathetic nerve activities have been primarily implicated in the development and maintenance of hypertension in both SHRs^[^ ^38^, ^39^, ^40^, ^41^ ^]^ and stress‐induced hypertensive rats.^[^ ^42^, ^43^, ^44^, ^45^ ^]^

**Figure 7:**
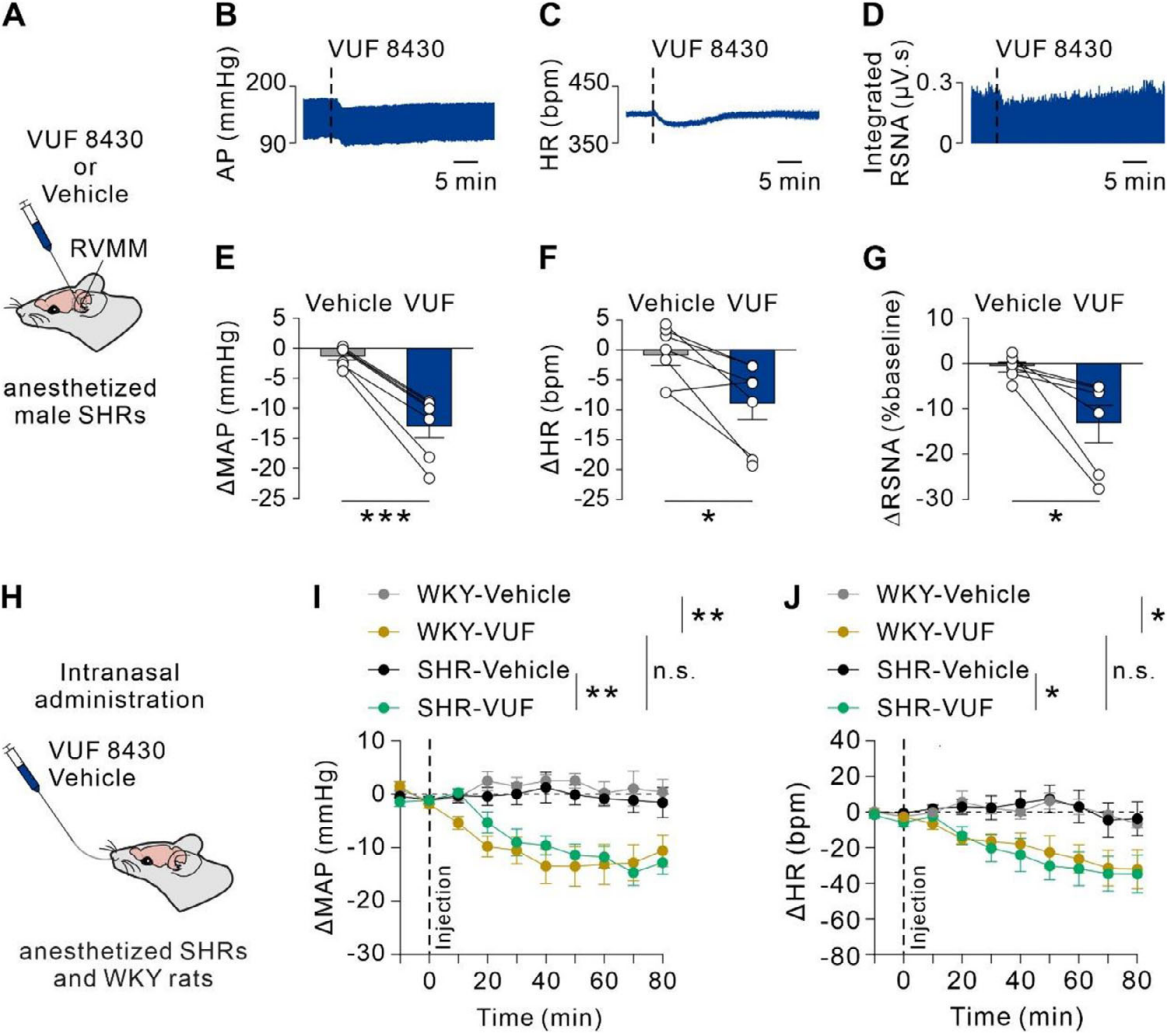
Activation of H4R in RVMM induced a comparable hypotensive effect on both anesthetized SHRs and normotensive WKY rats. A) Schematic of microinjection of selective H4R agonist VUF 8430 or vehicle into RVMM in anesthetized SHRs. B–D) Raw traces illustrating changes in MAP (B), HR (C), and integrated RSNA (D) induced by microinjection of VUF 8430 into RVMM of SHRs. (E‐G) Group data of maximal responses of MAP E), HR F), and integrated RSNA G) after microinjection of VUF 8430 or vehicle into RVMM of SHRs (E: *n* = 7; *P* = 0.0002; F: *n* = 7, *P* = 0.0148; G: *n* = 6, *P* = 0.0283; two‐tailed paired student's t‐test). H) Schematic of intranasal delivery experiments in anesthetized SHRs and normotensive WKY rats. I) Intranasal administration of VUF 8430 significantly decreased MAP in both anesthetized SHRs and normotensive rats (*n* = 5 for per group, interaction *P* < 0.0001; WKY‐Vehicle vs WKY‐VUF: *P* = 0.0034, SHR‐Vehicle vs SHR‐VUF: *P* = 0.0086, WKY‐VUF vs SHR‐VUF: *P* = 0.6344, repeated measures two‐way ANOVA with Bonferroni's multiple comparisons test). J) Intranasal administration of VUF 8430 significantly decreased HR in both anesthetized SHRs and normotensive rats (*n* = 5 for per group, interaction *P* < 0.0001; WKY‐Vehicle vs WKY‐VUF: *P* = 0.0140, SHR‐Vehicle vs SHR‐VUF: *P* = 0.0315, WKY‐VUF vs SHR‐VUF: *P* = 0.7359, repeated measures two‐way ANOVA with Bonferroni's multiple comparisons test). Group Data were presented as means ± S.E.M. *P < 0.05, **P < 0.01, ***P < 0.001, n.s. no significance.

In anesthetized SHRs, local microinjection of the selective H4R agonist VUF 8430 into the RVMM elicited pronounced cardiovascular responses, including significant reductions in MAP (12.93 ± 1.93 mmHg), HR (8.92 ± 2.66 bpm), and RSNA (13.36% ± 4.14%) compared with vehicle controls (**Figure** **7**A–G). Notably, these effects were consistent across both male and female SHRs (Figure S8, Supporting Information), indicating the absence of sex‐dependent differences in H4R‐mediated responses. Comparative analysis of the net cardiovascular responses to microinjection of VUF revealed no significant difference in the hypotensive effects of H4R activation between hypertensive and normotensive rat models (Figure S9, Supporting Information). Preservation of H4R functionality in hypertensive states was further supported by systemic administration studies, in which intranasal delivery of VUF 8430 produced comparable hypotensive and bradycardic effects in both normotensive and SHRs (Figure 7H–J). At the molecular level, quantitative analyses revealed no significant changes in H4R mRNA expression or histamine content in RVMM tissue between normotensive and hypertensive animals, including both SHRs and stress‐induced hypertensive rats (Figure S10, Supporting Information). These findings, spanning molecular to physiological levels, indicate that H4R‐mediated neuromodulation remains structurally and functionally intact during hypertension development. The consistent responses observed across administration routes, animal models, and sexes underscore that H4R operates as a stable neuromodulatory system rather than a primary pathogenic factor in hypertension.

Given the clinical relevance of intranasal delivery for brain‐targeted therapeutics,^[^ ^46^ ^]^ we systematically evaluated this route for H4R agonist administration. Pharmacokinetic analysis using high‐performance liquid chromatography‐mass spectrometry (LC‐MS) demonstrated rapid delivery of VUF 8430 (0.25 mg kg^−1^) to the RVMM, with peak concentrations observed 1 h post‐administration in SHRs and complete clearance within 24 h (Figure S11, Supporting Information), supporting the feasibility of once‐daily dosing for therapeutic purposes. The central mechanism was confirmed via pharmacological blockade: pretreatment with the selective H4R antagonist JNJ 10191584 in the RVMM abolished the cardiovascular effects of intranasal VUF 8430 in anesthetized SHRs (Figure S12, Supporting Information), indicating that these responses are predominantly mediated through H4R activation in the RVMM. Anatomical specificity was further supported by control experiments showing that RVLM microinjection of VUF 8430 did not alter cardiovascular parameters in SHRs (Figure S13A—C, Supporting Information), consistent with observations in normotensive animals. In addition, analysis of publicly available transcriptomic data from rat brainstem nuclei (GSE234784) showed negligible expression of *Hrh4* in both the nucleus of the tractus solitarius (NTS) and the caudal ventrolateral medulla (CVLM) (Figure S13D, Supporting Information), two major centers in central baroreflex pathways,^[^ ^6^ ^]^ indicating that cardiovascular regulation mediated by H4R occurs primarily in the RVMM rather than in the NTS or CVLM. Collectively, these data demonstrate that intranasal administration of H4R agonists can effectively target the RVMM to produce antihypertensive effects.

**Figure 8:**
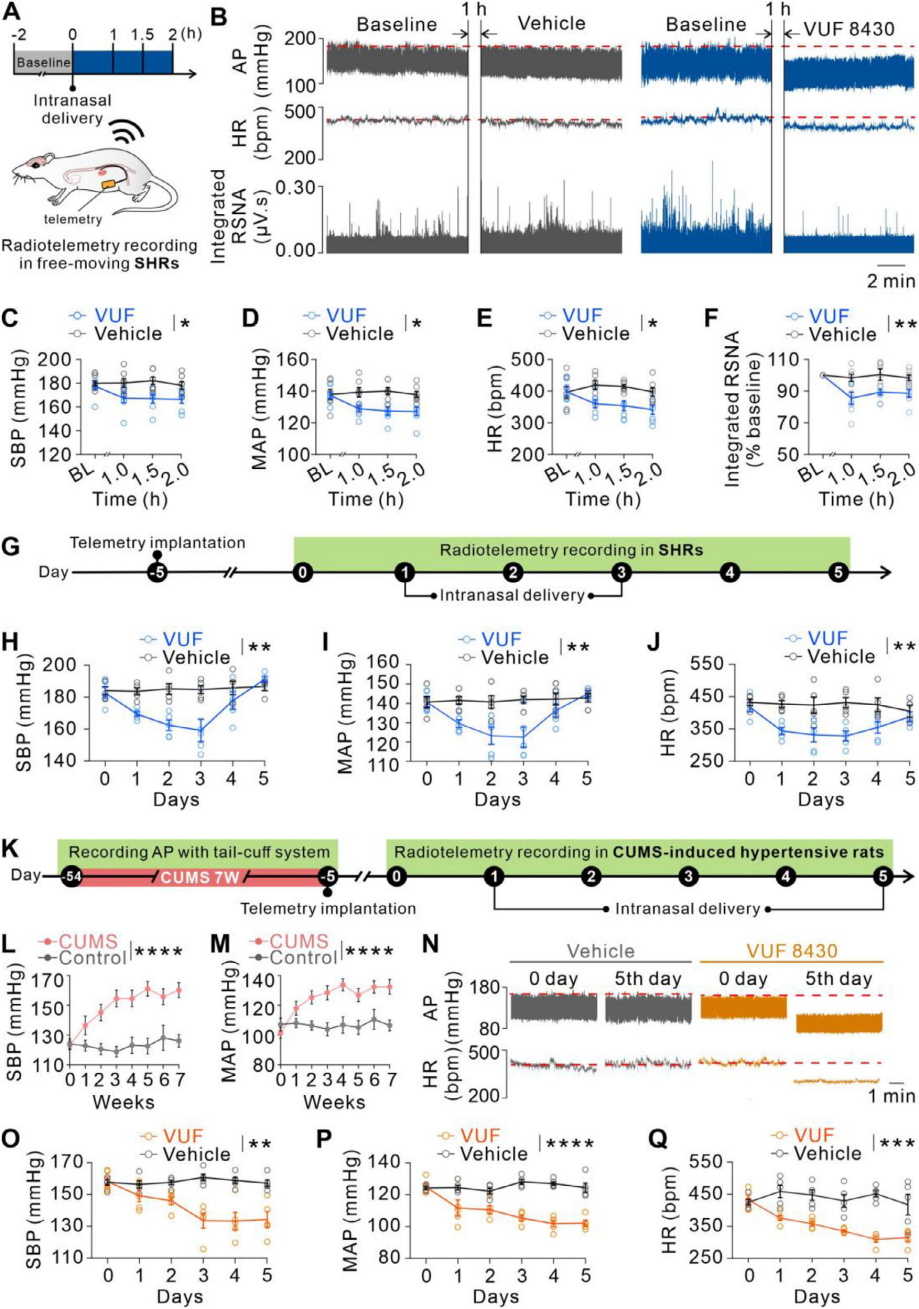
Intranasal delivery of VUF 8430 ameliorates hypertension in free‐moving SHRs and CUMS‐induced hypertensive rats. A) Scheme of the experimental paradigm showing acute intranasal drug delivery with telemetric monitoring of AP, HR, and RSNA in SHRs. B) Single‐dose intranasal VUF 8430 significantly decreased AP, HR, and integrated RSNA in free‐moving SHRs. C–F) Group data of SBP (C; *n* = 7, *P* = 0.0279), MAP (D; *n* = 7, *P* = 0.0164), HR (E; *n* = 7, *P* = 0.0276), and integrated RSNA (F; *n* = 7, *P* = 0.0032) recording before and after drug administration (repeated measures two‐way ANOVA with Bonferroni's multiple comparisons test). BL, baseline. G) Scheme of the experimental paradigm showing 3‐day intranasal drug delivery with telemetric monitoring of SBP, MAP, and HR. H–J) Group data of SBP (H; *n* = 5, *P* = 0.0015), MAP (I; *n* = 5, *P* = 0.0072), and HR (J; *n* = 5, *P* = 0.0020) changes for chronic administration of H4R agonist VUF 8430 and vehicle (mixed‐effects analysis with Bonferroni's multiple comparisons test). K) Scheme of the experimental paradigm showing intranasal drug delivery of H4R agonist VUF 8430 once a day for consecutive 5 days with telemetric monitoring of cardiovascular activities in hypertensive rats exposed to CUMS for 7 weeks. L and M) The SBPs (L) and MAPs (M) of CUMS and control group measured by tail‐cuff (CUMS: *n* = 10, Control: *n* = 8; *P* < 0.0001, repeated measures two‐way ANOVA with Bonferroni's multiple comparisons test). N) Raw tracings showed a significant reduction of AP and HR on the 5th day in CUMS‐induced hypertensive rats with intranasal delivery of VUF 8430. O–Q) Group data of changes in SBP (O; *n* = 5, *P* = 0.0027), MAP (P; *n* = 5, *P* < 0.0001), and HR (Q; *n* = 5, *P* = 0.0003) for chronic intranasal administration of VUF 8430 and vehicle (repeated measures two‐way ANOVA with Bonferroni's multiple comparisons test). Group Data were presented as means ± S.E.M. *P < 0.05, **P < 0.01, ***P < 0.001, ****P < 0.0001.

To assess the therapeutic potential of chronic H4R activation, we performed longitudinal cardiovascular monitoring using radiotelemetry in freely moving SHRs (**Figure** **8**A). A single intranasal dose of VUF 8430 significantly decreased systolic blood pressure (SBP), MAP, and HR, and suppressed RSNA in free‐moving SHRs, consistent with results obtained under anesthesia (Figure 8B–F). For chronic treatment evaluation, SHRs received once‐daily intranasal VUF 8430 for 3 consecutive days (Figure 8G). Continuous radiotelemetry recordings demonstrated a sustained decrease of SBP, MAP, and HR in the free‐moving SHRs followed by a recovery after drug withdrawal (Figure 8H–J).

Given that prolonged stress significantly contributes to the prevalence of hypertension in modern society,^[^ ^47^, ^48^ ^]^ we further determined the long‐term effect of intranasal delivery of VUF 8430 on cardiovascular activities in free‐moving hypertensive rats exposed to CUMS for 7 weeks (Figure 8K). Similar to previous studies,^[^ ^49^ ^]^ the SBPs and MAPs of CUMS‐induced hypertensive rats were significantly increased and reached a sustained high level (Figure 8L,M). We found that intranasal administration of VUF 8430 once a day for consecutive 5 days gradually and consistently reduced SBP, MAP, and HR of CUMS‐induced hypertensive rats (Figure 8N–Q). These results collectively suggest that histamine H4R may provide a potential central target for treating neurogenic hypertension.

## 3 Discussion

The central cardiovascular centers are mainly located in the medulla oblongata and hypothalamus, whose functional changes have been implicated in most forms of hypertension.^[^ ^50^, ^51^ ^]^ In the present study, we reveal that RVMM receives direct innervation from hypothalamic histaminergic neurons and can be modulated by histamine to produce depressor and bradycardia responses via TRPV1 coupled to histamine H4R. Furthermore, activating the H4R in RVMM potently ameliorates hypertension in SHRs and CUMS‐induced hypertensive rats.

As the newest member of the histamine receptor family, H4R has received great interest since it was identified by several independent research groups two decades ago.^[^ ^25^, ^26^ ^]^ However, H4R expression in the brain has not been convincingly reported until now. Insufficient quality of antibodies and uncritical interpretation of RT‐PCR results instead of qPCR experiments may be primarily responsible for the controversy.^[^ ^52^ ^]^ Here, by analysis of publicly available snRNA‐seq datasets of human brains, we reported a relatively higher expression of *HRH4* in neurons of the medulla oblongata. Furthermore, by the employment of RNA in situ hybridization combined with immunofluorescence integrated co‐detection, we identified for the first time that *Hrh4* is distributed in the RVMM and RVLM neurons. Moreover, qPCR results indicated a comparable level of *Hrh4* relative expression between RVMM and RVLM. Therefore, we demonstrate a specialized distribution of H4R in the RVMM and RVLM and its cardiovascular function. However, the current lack of validated antibodies precludes definitive protein‐level verification of H4R expression, which remains an important question for future studies.

Microinjection of VUF 8430, a selective agonist for H4R, into the RVMM produced a depressor and bradycardia response in this study. Moreover, we found that H4R activation induced a direct postsynaptic excitatory effect on RVMM neurons, indicating that histamine H4R is actively involved in the endogenous negative regulation of cardiovascular activity via exciting the depressor neurons located in RVMM. Early neuroanatomical and pharmacological studies have shown that the depressor zones are scattered over the RVMM^[^ ^53^, ^54^ ^]^ and probably innervate the sympathetic preganglionic neurons in the thoracic spinal cord via GABAergic efferent.^[^ ^55^ ^]^ Current studies have found an excitatory and inhibitory spinal projecting population in RVMM via an intraspinal injection of retrograde tracing virus in a transgenic mouse line.^[^ ^35^ ^]^ In this study, we observed that *Hrh4* was specifically distributed on GABAergic, but not glutamatergic, RVMM neurons that project transsynaptically to the kidney. Renal sympathetic nerve activation is thought to reduce glomerular filtration rate and renal blood flow via afferent arteriole constriction. In addition, it stimulates renin release from juxtaglomerular cells and enhances tubular sodium and water reabsorption, collectively promoting blood pressure elevation and contributing to hypertension pathogenesis.^[^ ^34^, ^56^ ^]^ Here we found that H4R activation‐excited RVMM neurons were GABAergic and induced inhibitory renal sympathetic outflow. We thus believed that the H4R activation in the RVMM may excite the direct GABAergic descending pathways to spinal sympathetic preganglionic neurons, which leads to an inhibition of sympathetic activity and subsequently lower blood pressure and HR.

Although sympathetic overactivity modulated by H4R represents a hallmark feature of refractory hypertension,^[^ ^3^ ^]^ our experimental evidence suggests that H4R does not directly drive hypertension pathogenesis, yet remains a promising therapeutic target. Notably, we observed no significant changes in H4R expression or histamine levels in either SHRs or stress‐induced hypertensive rats compared with normotensive controls, although both hypertensive models exhibited a trend toward elevated histamine levels. This observation, together with the robust hypotensive effects of H4R activation demonstrated in our study, suggests that increased histamine may act as a compensatory mechanism to counteract hypertension progression. Furthermore, pharmacological activation of H4R with VUF8430 produced comparable hypotensive responses in normotensive and hypertensive rats, with no sex‐dependent differences, indicating that receptor functionality is preserved under hypertensive conditions. Collectively, these findings demonstrate that H4R maintains functional integrity during the development of hypertension, functioning as a stable neuromodulatory system rather than a primary pathogenic factor. This distinction is clinically relevant given the multifactorial etiology of hypertension, where genetic, physiological, and environmental interactions converge to drive sympathetic overactivity, a defining characteristic of refractory hypertension.^[^ ^1^, ^3^, ^5^ ^]^ The therapeutic potential of H4R modulation is fully supported by its ability to reliably suppress sympathetic outflow irrespective of hypertension etiology, consistently reduce blood pressure across varying hemodynamic states, and specifically target RVMM GABAergic presympathetic neurons to address the sympathetic overactivity underlying refractory hypertension.

Unexpectedly, although histamine H4R is also expressed in the RVLM, its activation failed to elicit any detectable cardiovascular effects in either normotensive conditions or SHRs. This is most likely because H4R activation did not influence the excitability of RVLM neurons. Indeed, the H4R‐mediated regulation of neuronal excitability has not yet been reported in other brain regions. However, in the peripheral nervous system, H4R activation has been found to excite DRG neurons via the opening of TRPV1 channels.^[^ ^32^, ^57^ ^]^ Interestingly, blockage of TRPV1 channels significantly diminished the excitation on RVMM neurons induced by H4R activation and the depressor and bradycardia response evoked by optogenetic activation of histaminergic afferent terminals in RVMM, indicating that TRPV1 channels may be coupled to H4R and mediate the excitatory effect of H4R activation on RVMM neurons and the consequent cardiovascular regulation. Although TRPV1 channels are also expressed in RVLM neurons, they were not co‐localized with H4R on the same RVLM neurons. This may be the reason why H4R activation cannot excite RVLM neurons and affect RVLM‐mediated cardiovascular regulation. Considering that activation of H4R has been reported to result in stimulating the mitogen‐activated protein kinase (MAPK) pathways and inhibiting the cAMP‐PKA‐CREB signaling pathway,^[^ ^58^ ^]^ whether H4R activation in the RVLM may recruit these downstream signaling cascades still needs to be further elucidated.

The histamine receptor family is one of the most rewarding families of drug targets to date.^[^ ^13^ ^]^ H1‐, H2‐, and H3‐antihistamines have been widely applied in the clinical treatment of allergies, ulcers, and vestibular disorders.^[^ ^13^, ^17^ ^]^ Although H1, H2, and H3 receptors are also expressed in the myocardial and vascular tissues,^[^ ^59^ ^]^ their extensive distribution in the various peripheral tissues and brain regions results in the complexity of their cardiovascular effects and the variety of side effects. It has been reported that overdoses of antihistamines may produce undesirable side effects in cardiovascular physiology. Most patients with an overdose of diphenhydramine, an H1 antihistamine, manifest hypertension and tachycardia.^[^ ^60^ ^]^ In normotensive subjects, sustained postexercise vasodilatation is abolished by combined oral H1R and H2R antagonists, fexofenadine and ranitidine.^[^ ^61^ ^]^ The incidence of cardiovascular‐related treatment‐emergent adverse events, including tachycardia and increased heart rate, appears to exhibit a dose‐related trend in the patients treated with bavisant, a highly selective H3R antagonist.^[^ ^62^ ^]^ Therefore, H1R, H2R, and H3R may not be promising targets for cardiovascular diseases. In the present study, we found that blockade of H4R and its downstream TRPV1 in RVMM effectively abolished the depressor and bradycardia response induced by optogenetic activation of TMN‐RVMM histaminergic afferents, suggesting a great contribution of H4R to the histamine‐mediated antihypertensive effect on RVMM. Furthermore, we observed that activating histamine H4R rather than H1R, H2R, and H3R in RVMM remarkably elicited depressor and bradycardia responses and a decreased RSNA. Therefore, the rare distribution of H4R in widespread brain areas and the well‐defined expression and cardiovascular function of H4R in RVMM suggest that non‐invasive drug delivery targeting H4R in RVMM may be an effective strategy for treating hypertension.

H4R was initially identified in the peripheral immune system, with variable expression in the bone marrow, spleen, and gastrointestinal tract.^[^ ^13^ ^]^ This distribution necessitates careful consideration of potential peripheral effects when developing central nervous system‐targeted H4R therapies, particularly given its roles in immunomodulation, allergic responses, gastrointestinal inflammatory processes, pruritus, and nociceptive signaling.^[^ ^22^, ^24^ ^]^ In the present study, we employed intranasal delivery of an H4R agonist to minimize peripheral exposure while directly targeting the brain, circumventing the widespread distribution associated with oral or intraperitoneal administration. This approach effectively limited peripheral effects while achieving robust antihypertensive outcomes in both SHRs and CUMS‐induced hypertensive models following VUF 8430 administration. Pharmacokinetic analyses confirmed rapid RVMM penetration, with detectable levels at 1 h and complete clearance within 24 h. The complete abolition of cardiovascular responses following selective RVMM H4R blockade validates this nucleus as the primary site of action, whereas other cardiovascular centers likely exhibit either lower H4R expression or insufficient downstream signaling components, such as TRPV1. Given that H4R in the RVMM plays a specific role in central cardiovascular regulation, while other histamine receptor subtypes are widely distributed in the brain and periphery to mediate diverse physiological functions, we suggest that RVMM H4R may provide a potential target for the treatment of hypertension with few side effects in neurogenic hypertension.

In summary, we demonstrate that activating histamine H4R in RVMM produces depressor and bradycardia responses by exciting GABAergic RVMM presympathetic neurons by opening TRPV1 channels. The findings identify a significant antihypertensive effect of H4R agonism in the SHR and CUMS‐induced hypertensive rats, and suggest a potential therapeutic target for hypertension, particularly neurogenic and sympathetically driven forms.

## 4 Experimental Section

### Animals

Wildtype Sprague‐Dawley rats (6–8 w) were obtained from the Experimental Animal Center of Nanjing Medical University. SHRs (10–12 w) were purchased from Charles River Laboratories (China). The HDC‐Cre rats (6–8 w) were generated using CRISPR/Cas9 technology as we previously reported.^[^ ^15^ ^]^ The *Hrh4*‐KO mice were purchased from Shanghai Model Organisms Center (China). All animals were individually housed under controlled environmental conditions (22 ± 2 °C; 60% ± 5% humidity; and 12 h light/dark cycle with lights on at 9:00 a.m. daily).

The CUMS‐induced hypertensive rats were produced, as reported previously.^[^ ^37^ ^]^ In brief, adult male Sprague‐Dawley rats were exposed to CUMS for 7 weeks, including water or food deprivation for 12 h, reversed light/dark cycle for 24 h, physical restraint for 2 h, a 45⁰ cage tilt for 2 h, a crowded cage for 2 h, forced swimming for 3 min. These stressors were applied 2 times every day. The rats were exposed to different stressors at random every day, making it impossible for the animals to predict the stimulus. The same stressor was not applied on consecutive days. We used a noninvasive tail‐cuff system (BP‐300A, TECHMAN, China) for daily monitoring of blood pressure in CUMS and control groups. Tail artery blood pressure was obtained by averaging 10–15 measurements each day. Blood pressure elevation was observed in CUMS group and remained for at least 2 weeks after termination of CUMS procedure. All efforts were taken to minimize the number of animals used and their suffering.

Rats were randomized for control or drug treatment. Investigators were blinded to rat treatment groups. All experimental protocols were carried out after approval by the Animal Ethical and Welfare Committee of Nanjing University and in compliance with the U.S. National Institutes of Health Guide for the Care and Use of Laboratory Animals. All efforts were taken to minimize the number of animals used and their suffering.

### Analysis of Human Single Nucleus RNA Sequencing Data

Published single‐nucleus RNA sequencing data from Human Brain Cell Atlas v1.0 was downloaded from CZ CELLxGENE (https://cellxgene.cziscience.com/collections/283d65eb‐dd53‐496d‐adb7‐7570c7caa443).^[^ ^30^ ^]^ A sub‐dataset for the dissection of the myelencephalon (medulla oblongata, Mo) was obtained for analysis. Nuclei were clustered and visualized based on the presence of well‐established cell types and UMAP projection by using the R package (R Foundation for Statistical Computing, Austria) and Seurat (Version 5.0.3). The expression of *HRH4* was analyzed and visualized using scCustomize (Version 2.1.2) and ggplot2 (Version 3.5.0). Analysis of *HRH4* with more than one read was determined as a high‐level expression.

### Analysis of Transcriptomic Data From Rat Brainstem Nuclei

Raw counts was analyzed of *Hrh4* expression in NTS and CVLM using publicly available sequencing data from rats (GSE234784).

### RNA‐protein integrated co‐detection

Adult male Sprague‐Dawley rats were anesthetized with sodium pentobarbital (40 mg kg^−1^; Sigma, USA) and perfused transcardially with normal saline and 4% paraformaldehyde (PFA) in 0.1 m phosphate buffer (PB). Then, the rat brain was removed, trimmed, post‐fixed in 4% PFA overnight at 4 °C, and cryoprotected with 20% sucrose and 30% sucrose for 24 h. Then, the formalin‐fixed frozen tissue sections of ≈20 µm thickness were mounted on adhesion microscope slides (Citotest, China). RNA‐protein integrated co‐detection was performed using RNA‐Protein Co‐detection Ancillary Kit (Advanced Cell Diagnostics, USA; Cat No. 323180) and RNAscope Multiplex Fluorescent Reagent Kit 2.0 (Cat No. 323100). In brief, the slides were post‐fixed in 4% PFA at 4 °C for 15 min, dehydrated with 50%, 70%, and 100% ethanol solutions step by step, followed by endogenous enzyme blocking with RNAscope hydrogen peroxide at room temperature (RT) for 10 min. Permeabilization was performed by boiling in a pressure cooker for 5 min in 1× Co‐detection Target Retrieval solution. Afterward, the slides were incubated with the primary antibody diluted in Co‐detection Antibody Diluent at 4 °C overnight. Following primary antibody incubation, all sections were submerged in 4% PFA for post‐primary fixation 30 min at RT. Protein digestion was achieved with the help of RNAscope Protease IV for 30 min at 40 °C. All steps at 40 °C were performed in a HybEZ oven (Advanced Cell Diagnostics) that ensures quick heating up of the samples. *Hrh4* probes (RNAscope Probe‐ Rn‐Hrh4; Cat No. 559161) were then hybridized at 40 °C for 2 h. To ensure that there was no background staining related to the RNAscope assay, a negative control probe (RNAscope 3‐plex Negative Control Probe; Cat No. 320871) was used. After hybridization, brain sections were sequentially applied with a series of probe signal amplification and fluorescence labeling steps according to the manufacturer's instructions. Finally, fluorophore‐conjugated secondary antibody diluted in Co‐Detection Antibody Diluent was added, and the slides were incubated for 30 min at RT. Finally, the slides were mounted in DAPI Fluoromount‐G mounting medium (Southern Biotech, USA; Cat No. 0100–20).

Primary and secondary antibodies used were as follows: rabbit anti‐NeuN polyclonal antibody (1:200; Proteintech Group, USA; Cat No. 26975‐1‐AP), rabbit anti‐TRPV1 polyclonal antibody (1:200; Gene Tex, USA; Cat No. GTX10296), rabbit anti‐GAD 65/67 polyclonal antibody (1:100; Sigma; Cat No. G5163), rabbit anti‐Glutamate polyclonal antibody (1:100; Sigma; Cat No. G6642) and donkey anti‐rabbit AlexaFluor 647 (1:1000; Invitrogen, Canada; Cat No. 150075). All pictures of representative areas of each slide were taken with the Zeiss LSM 880 confocal laser scanning microscope (Zeiss, Germany) at 40× magnification. Digital images from the microscope were recorded with Zen 2 Software (Zeiss).

### Real‐Time Fluorescence Quantification PCR (RT‐qPCR)

Adult male rats and *Hrh4*‐KO mouse were anesthetized with sodium pentobarbital (40 mg kg^−1^) and perfused transcardially with normal saline before removal of the brain. According to the rat brain atlas, the RVMM and RVLM tissues were collected from coronal brain slices.^[^ ^63^ ^]^ Total mRNA was extracted according to the manufacturer's instructions of the Trizol reagent (Vazyme, China) and subsequently transcribed into cDNA with the Hifair II 1st Strand cDNA Synthesis SuperMix kit (Yeasen, China). *Hrh4* expression was detected using standard protocols of Hieff UNICON qPCR SYBR Green Master Mix (Yeasen) and Light Cycler 480 real‐time RT‐PCR platform (Roche, Switzerland). The quantity of *Hrh4* was expressed relative to the amount of the reference gene glyceraldehyde‐phosphate dehydrogenase (*Gapdh*) to obtain a normalized target expression value.^[^ ^64^ ^]^ The corresponding Oligo (DT) primers for qPCR amplification were as follows:

(1) primers for rats

*Gapdh*‐forward: 5′‐TTCAACGGCACAGTCAAGG‐3′;

*Gapdh*‐reverse: 5′‐CTCAGCACCAGCATCACC‐3′;

*Hrh4*‐forward: 5′‐ATGTCGGAGTCTAACGGCAC‐3′;

*Hrh4*‐reverse: 5′‐ATGACACCCACGAAGAAGTCAG‐3′;

(2) primers for mice

*Gapdh*‐forward: 5′‐AGGTCGGTGTGAACGGATTTG‐3′;

*Gapdh*‐reverse: 5′‐TGTAGACCATGTAGTTGAGGTCA‐3′;

*Hrh4*‐forward: 5′‐TAGCCTTTGTGGTGGACAGA‐3′;

*Hrh4*‐reverse: 5′‐TGAGCCCTATAAGACACAGCAT‐3′.

### Immunohistochemistry

Immunohistochemistry was performed as we reported previously.^[^ ^15^, ^65^ ^]^ Frozen coronal slices (35 µm in thickness) containing TMN and RVMM were rinsed with 1 × PBST and incubated in 10% normal bovine serum in PBST for 30 min. Afterward, the slices were incubated overnight at 4 °C with primary antibodies and in the related secondary antibodies for 4 h at room temperature in the dark. The slides were washed and mounted in DAPI Fluoromount‐G mounting medium. Incubations replacing the primary antiserum with control immunoglobulins and/or omitting the primary antiserum were used as negative controls.

For immunohistochemical identification of the recorded GABAergic and glutamatergic RVMM neurons, brain slices containing biocytin‐filled neurons in the whole‐cell patch clamp recordings were fixed, dehydrated, re‐sectioned, and then incubated with GAD 65/67 or glutamate polyclonal primary antibody. All micrographs were obtained by a Zeiss LSM 880 confocal laser scanning microscope, and digital images from the microscope were recorded with Zen 2 Software. Primary antibodies used in the experiments: rabbit anti‐HDC antibody (1:200; Progen, Germany, Cat No. 16045), rabbit anti‐GAD65/67 polyclonal antibody (1:200; Sigma, Cat No. G5163), mouse anti‐glutamate monoclonal antibody (1:500; Millipore, USA, Cat No. MAB5304). Secondary antibodies used in the experiments: secondary antibodies (1:1000; Invitrogen) conjugated to Alexa 568 (Cat No. A‐11004), AlexaFluor 488 (Cat No. S11223), and AlexaFluor 647 (Cat No. 150075).

### Stereotaxic microinjection and implantation

Anesthetized rats were mounted on a stereotaxic frame (David Kopf Instruments, USA; 1404) for drug and virus microinjection and guide tube implantation. All coordinates were measured according to the rat brain atlas.^[^ ^63^ ^]^

For in vivo drug microinjection into RVMM and RVLM, the craniotomy was made above RVMM or RVLM. A Hamilton syringe was inserted to RVMM (AP – 11.52 mm, ML ± 0.8 mm, DV – 8.5 mm) or RVLM (AP – 12.12 mm, ML ± 2.4 mm, DV – 8.5 mm) for unilateral microinjection (0.5 µL each side, lasting 2 min) following drugs, as previous reported:^[^ ^66^, ^67^, ^68^, ^69^, ^70^ ^]^ selective H4R agonist VUF 8430 (8 µg; Tocris, UK, Cat No. 2494), selective H1R agonist 2‐pyridylethylamine (2‐PyEA) (1 µg; Tocris, Cat No. 2478), selective H2R agonist dimaprit (1 µg; Tocris, Cat No. 0506), selective H3R agonist (R)‐(‐)‐α‐Methylhistamine (RAMH) (1.4 µg; Tocris, Cat No. 0569) and vehicle (ACSF).

For optogenetic manipulation, we performed adeno‐associated vector (AAV) virus microinjection and guide tube implantation in HDC‐Cre rats. At day 1, 0.5 µL of AAV2/9‐hEF1α‐DIO‐ChR2‐EYFP or AAV2/9‐hEF1α‐DIO‐ EYFP (BrainVTA, China, Cat No. PT‐0001/PT‐0013) was bilaterally infused to hypothalamus TMN (AP – 4.5 mm, ML ± 1.2 mm, DV – 9.4 mm). Following viral injection, needles were kept at the injection site for 10 min to allow for viral diffusion. On the 18th day after the virus injection, two stainless‐steel guide tubes (length 11 mm, o.d. 0.8 mm, i.d. 0.5 mm) for optical fibers insert and antagonist infusion were bilaterally implanted 5.0 mm above RVMM of each animal. Rats were caged individually and allowed to recover for at least 72 h before cardiovascular activity recording.

The effective extent of the drug and virus diffusion in the present study was restricted in the microinjection sites according to the estimate by extracellular electrophysiological recording units 0.5–2.0 mm away from the injection site as per our previous report.^[^ ^71^ ^]^ Data from rats where the injection sites were histologically identified to deviate from the microinjection sites were excluded from further analysis (Figure S14, Supporting Information).

### Transsynaptic Retrograde Tracing by PRV Injections Into the Kidney

After anesthesia by intraperitoneal injection of sodium pentobarbital (40 mg kg^−1^), the left kidneys of adult male Sprague‐Dawley rats were exposed retroperitoneally. PRV (BrainCase, China; Cat No. BC‐PRV‐531‐Plus) were injected at the rostral, middle, and caudal parts of the left renal parenchyma using a glass micropipette (0.8 µL per site). To limit the spread of PRV and prevent bleeding, the micropipette was kept in situ for 10 min after every injection.^[^ ^72^ ^]^ 4 days after PRV injection, all rats were re‐anesthetized and perfused transcardially with normal saline and 4% PFA to prepare brain slides for RNA‐protein integrated co‐detection.

### Whole‐Cell Patch Clamp Recording

Whole‐cell patch clamp was performed as previously described.^[^ ^73^, ^74^ ^]^ Rats and *Hrh4*‐KO mice of both sexualities aged postnatal days 12–16 were employed. Coronal brain stem slices (300 µm thick) containing TMN, RVMM, and RVLM were prepared with a vibroslicer (Leica, Germany; VT 1200 S). The slices were incubated in ACSF containing in mM: 124 NaCl, 2.5 KCl, 1.25 NaH_2_PO_4_, 1.3 MgSO_4_, 26 NaHCO_3_, 2 CaCl_2_, and 20 d‐glucose, at 35 °C for at least 1 h and then maintained at room temperature. RVMM and RVLM neurons were visualized with an Olympus BX51WI microscope equipped with infrared differential interference contrast. Whole‐cell patch‐clamp was acquired with an Axopatch‐700B amplifier (Axon Instruments, USA), and the signals were fed into the computer through a Digidata‐1440A interface (Axon Instruments), and the data were analyzed by software pClamp 10.5 (Axon Instruments).

Neurons were recorded with borosilicate glass pipettes (3–5 MΩ) filled with an internal solution (composition in mM:140 K‐methylsulfate, 7 KCl, 2 MgCl_2_, 10 HEPES, 0.1 EGTA, 4 Na_2_‐ATP, 0.4 GTP‐Tris, and 4% biocytin adjusted to pH 7.25 with 1 m KOH). The whole‐cell current or membrane potential of the recorded neuron was observed for at least 20 min to ensure stability. Then selective H4R agonist VUF 8430 (3–300 µM) or histamine (0.3–30 µM; Tocris, Cat No. 3545) were added in the perfusing ACSF to stimulate the recorded neuron for 1 min. After each stimulation, cells were given at least 20 min for recovery and prevention of desensitization. The selective H4R antagonist JNJ 10191584 (3–30 µM; Tocris, Cat No. 2441) was applied to block the effect of VUF 8430 and histamine. TRPV1 antagonist AMG 9810 (30 µM; Abcam, UK, Cat No. ab145874) was applied to block the effect of VUF 8430. TTX (0.3 µM; Tocris, Cat No. 1069), AMPA receptor antagonist NBQX (30 µM; Tocris, Cat No. 0373), NMDA receptor antagonist AP5 (50 µM; Tocris, Cat No. 0106), and GABA_A_ receptor antagonist SR 95531 (50 µM; Tocris, Cat No. 1262) were used to block the synaptic transmission. The receptor antagonist or ion channel blockers were perfused for at least 15 min to ensure their effectiveness. Light‐evoked action potentials in the ChR2‐positive TMN neurons were recorded using a potassium‐gluconate‐based internal. Optostimulation was delivered as 470 nm light pulses (1, 5, 10, 15, and 20 Hz; 10‐ms pulse width) via a CoolLED system (pE‐300white, CoolLED, Andover, UK) mounted on an upright microscope. The maximal light output was 2 mW, as measured with an optical power meter (Thorlabs, Newton, NJ, USA).

### Optogenetic manipulation

For optogenetic activation of TMN‐RVMM histaminergic terminals, the optical fibers (200 µm core, NA = 0.37; Newdoon, China) were inserted to protrude 5 mm beyond the tip of the guide tubes aimed over RVMM of HDC‐rats. To permit bilateral manipulation, the single end of 2 × 1 fiber splitter (Newdoon) was connected to a rotating commutator (Doric, Canada), which was then attached via a fiber to a laser (Newdoon). Light output was measured with an optical power meter and adjusted to 60 mW of 473 nm light. We applied 473 nm light stimulation at 1, 5, 10, 15, and 20 Hz for 15 min. For blocking the cardiovascular effects elicited by opto‐stimulation, JNJ 10191584 (10 µM), AMG 9810 (30 µM), or vehicle (0.01% DMSO in ACSF) were infused into RVMM 5 min before light using Hamilton syringes (0.5 µL each side, lasting 2 min) via guide tubes.

### Cardiovascular activity recording in vivo

Cardiovascular activity recording in vivo was performed as we reported previously.^[^ ^75^ ^]^ Rats were anesthetized with isoflurane and maintained at surgical depth throughout the experimental session. Rectal temperature was maintained at 37 ± 0.5 °C. The trachea was intubated for artificial respiration. The left carotid artery was cannulated with polyethylene tubing (i.d. 0.5 mm, o.d. 0.9 mm) filled with normal saline containing heparin (30 U ml^−1^; Sigma) to measure AP. The polyethylene tubing was connected to a physiological pressure amplifier (Bridge Amp; AD Instruments, Australia) through a transducer (SP844; AD Instruments). MAP and HR were derived from the AP tracing. All signals were acquired online using LabChart 7 software.

The left kidney was exposed retroperitoneally, and the renal nerve branch, generally found in the aortic‐renal artery angle, was isolated, cut distally, and placed on bipolar silver wire electrodes to permit the recording of RSNA. The renal nerve branch and the electrodes were immersed in warm liquid paraffin. RSNA signals from the recording electrodes were amplified (×10k), filtered (bandwidth 100–2000 Hz), and recorded on a computer using a PowerLab system (AD Instruments). LabChart 7 software was used to rectify and integrate the renal sympathetic nerve signals. At the end of the experiment, the central end of the renal sympathetic nerve was cut to eliminate renal sympathetic efferent activity for electrical noise recording. The actual RSNA integral was determined by subtracting the noise integral from the recorded RSNA integral. The percentage relative to the baseline of integrated RSNA was analyzed.

### Telemetry Transmitter Implantation and Cardiovascular Activity Measurements

The cardiovascular activities of conscious, free‐moving SHRs and CUMS‐induced hypertensive rats were monitored by a telemetry system (TRM56SP; AD Instruments) and LabChart software. All rats were anesthetized by sodium pentobarbital (40 mg kg^−1^), and the femoral artery was surgically exposed and isolated. Then, the sensor for AP recording was connected to a telemetry transmitter, cannulated into the femoral artery, and forwarded to the abdominal aorta. The body of the transmitter was put into a subcutaneous pocket along the flank. Next, the electrode wire leads for RSNA recording were tunneled through to an incision on the left flank. The left kidney was exposed retroperitoneally, and the renal nerves, gently freed from surrounding connective tissue, were placed over the bipolar recording electrodes. Both electrodes and nerves were isolated with a biocompatible silicone elastomer (Kwik‐Sil; WPI, USA) and further secured with tissue adhesive. After recovery for 5 days, cardiovascular recording and drug treatment were performed in free‐moving SHRs and CUMS‐induced hypertensive rats. VUF 8430 (0.25 mg kg^−1^) or vehicle (ACSF) was intranasally administrated in SHRs for 3 consecutive days and in hypertensive rats for 5 consecutive days. Biological signals monitored continuously 10:00 a.m–18:00 p.m. were calculated and used for data analysis.

### Intranasal Delivery

Intranasal administration was performed both during acute cardiovascular recordings in anesthetized SHRs and during long‐term radiotelemetry monitoring in free‐moving rats, following pervious established protocols.^[^ ^76^, ^77^ ^]^ Rats received VUF 8430 (0.25 mg kg^−1^) or vehicle (ACSF) using a 25‐uL Hamilton syringe connected to a 15‐cm polyethylene tubing (i.d. 0.5 mm, o.d. 0.9 mm). The drug solution was distributed equally at the tip of polyethylene tubing and allowed to diffuse in the squamous epithelium of both the left and right rhinarium, whereas direct application into one of the nostrils was avoided. For intranasal delivery in freely moving rats, animals were lightly anesthetized with isoflurane to minimize struggling and stress. To block cardiovascular effects induced by intranasal VUF 8430, 30 µM JNJ 10191584 was infused into the RVMM 5 min prior to administration (1 µL per side over 2 min) via Hamilton syringes through guide tubes.

### High‐Performance Liquid Chromatography‐Mass Spectrometry (LC‐MS)

RVMM tissues were collected from all rats to determine histamine and VUF 8430 concentrations. Tissues were homogenized in precooled methanol/acetonitrile/water (2:2:1), followed by ultrasonic extraction for 30 min, centrifugation to collect the supernatant, and vacuum concentration to dryness. Samples were then reconstituted in 200 µL of 50% methanol for LC‐MS analysis.

All samples were separated by using the Shimadzu *Nexera X2* LC‐30AD ultra‐high performance liquid chromatography system (Shimadzu, Japan). The mobile phase consisted of water containing 0.1% formic acid (A) and acetonitrile containing 0.1% formic acid (B). The column temperature was maintained at 40 °C. The flow rate of the mobile phase was set to 300 µL min^−1^, and the injection volume was 1 µL. The gradient elution program was as follows: 0–17.5 min, 3%–70% B; 17.5–18 min, 70%–95% B; 18–20 min, 95% B; 20‐20.5 min, 95%–3% B; 20.5–23 min, 3% B. The mass spectrometry analysis was conducted using the 5500 QTRAP mass spectrometer (AB SCIEX) in the negative ion mode: source temperature 550 °C, ion source gas 1 (GS1): 55, ion source gas 2 (GS2): 55, curtain gas (CUR): 35, ion spray voltage (IS) 5500 V. The mass spectrometer was operating in multiple reaction monitoring (MRM) mode.

The chromatographic peak areas and retention times were extracted using Analyst 1.6.3 software. Quantitative analyses were conducted for all samples based on the retention time and peak shape information of the standard substances. The peak area of each chromatographic peak represents the relative content of the corresponding substance. These values were substituted into the linear equation and calculation formula, and the final quantitative analysis results of the substances to be detected in all samples were obtained.

### Statistics

All data were analyzed by GraphPad Prism 8.0 and presented as mean ± S.E.M. Statistical significance of RT‐qPCR was assessed by two‐tailed unpaired student's t‐test. *P* values of MAP, HR, and integrated RSNA changes in cardiovascular recording under anesthesia were determined by two‐tailed paired student's t‐test. And inward current changes in patch‐clamp recording were determined by two‐tailed paired student's t‐test and repeated measures one‐way ANOVA with Bonferroni's multiple comparisons test. Time‐course changes in SBP, MAP, HR, and integrated RSNA were analyzed by repeated measures two‐way ANOVA or mixed‐effects analysis with Bonferroni's multiple comparisons test. Concentration of histamine and VUF 8430 levels in RVMM were analyzed by repeated measures one‐way ANOVA with Bonferroni's multiple comparisons test. Percentages of immunoreactive colocalized cells were analyzed by a two‐tailed unpaired student's t‐test. The net cardiovascular responses resulting from H4R activation were determined by subtracting the vehicle‐induced responses from those elicited by the H4R agonist VUF in each paired experiment. A *P*‐value < 0.05 was considered statistically significant. A comprehensive summary of baseline hemodynamics of all experiments done under anesthesia was presented in the Supporting Information Table S1 (Supporting Information). The details of statistical analysis were supported in the Supporting Information Table S2 (Supporting Information) and the Supporting Data file.

## Conflict of Interest

The authors declare no conflict of interest.

## Supporting information



Supporting Information

Supporting Information

## Data Availability

The data that support the findings of this study are available in the supplementary material of this article.
