# Supplementary material for: Targeting Histamine H4 Receptor in the Rostral Ventromedial Medulla to Relieve Hypertension
Source: Adv Sci (Weinh). 2025 Sep 29;12(45):e08176. doi: 10.1002/advs.202508176 (PMC12677622; doi:10.1002/advs.202508176)
Supplement: Supplementary file 1 — Supporting Information [file ADVS-12-e08176-s001.docx]

Supporting Information

**Targeting histamine H4 receptor** **in the rostral ventromedial medulla to relieve hypertension**

*Ying Shi, Yang-Xun Zhang, Jun-Yi Chen, Sai Ma, Wei-Xuan Xue, Wei Li, Qian-Xiao Li, Bo Song, Ya-Ting Li, Hong-Yu Ma, Shu-Tao Xie, Hong-Zhao Li, Di-Jun Chen, Qi-Peng Zhang, Hui-Jie Ma, Jian-Jun Wang, Lei Yu*, Xiao-Yang Zhang*, Jing-Ning Zhu**

**
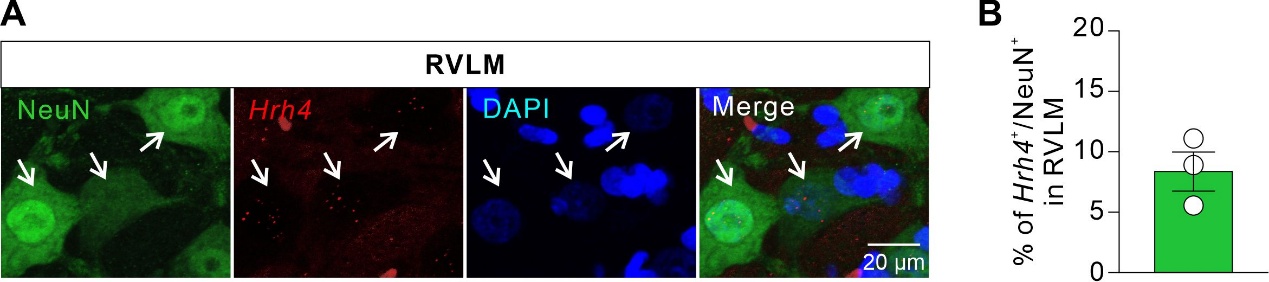
**

**Figure S1. H4R is expressed in RVLM.** (**A**) In situ hybridization combined with immunohistochemistry staining showed that H4R mRNA (*Hrh4*, red) localized on neurons (NeuN, green) in RVLM. Arrows showed RVLM neurons expressing H4R mRNA. Scale bar, 20 µm. (**B**) Percentage of neurons expressing *Hrh4* in RVLM (*n* = 3). Group data were presented as means ± S.E.M.


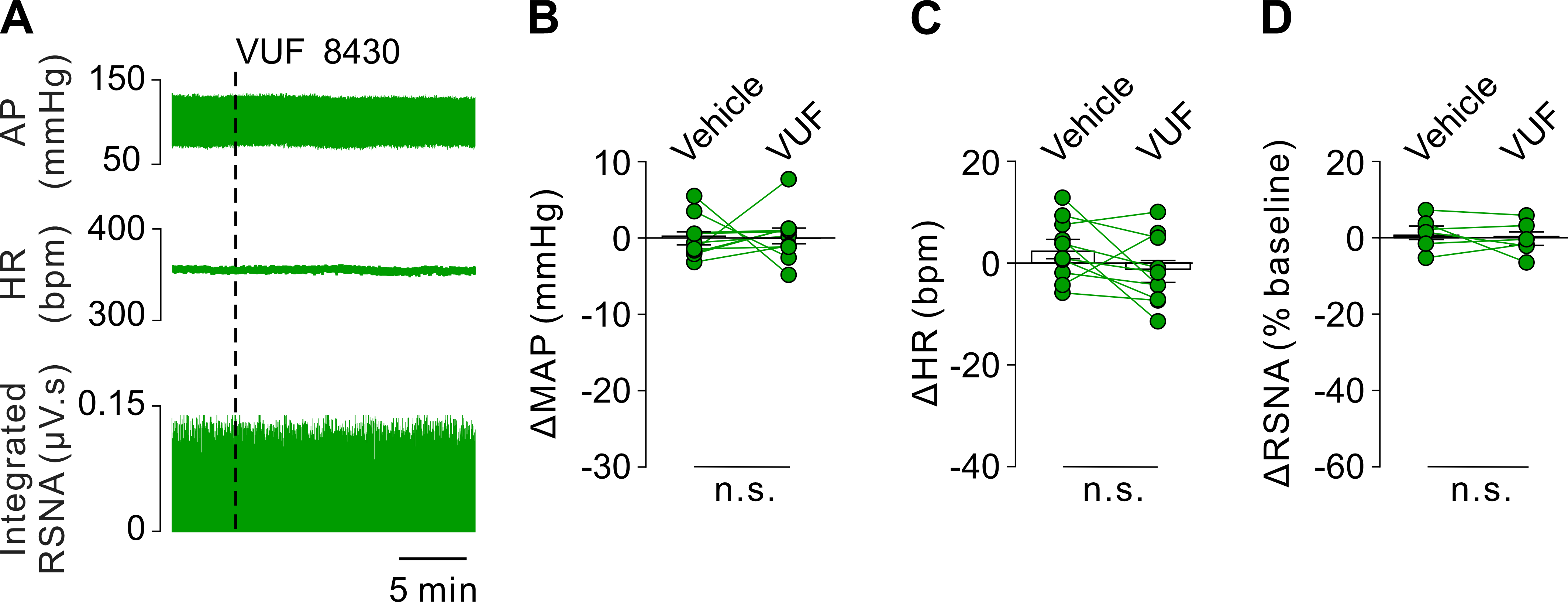


**Figure S2. Activation of H4R in RVLM does not affect MAP, HR, and RSNA in normal rats *in vivo*.** (**A**) Raw traces illustrating changes in AP, HR, and RSNA induced by microinjection of VUF 8430 into RVLM. (**B**-**D**) Group data of maximal responses of MAP, HR, and RSNA after microinjection of VUF 8430 or vehicle into RVLM (B: *n* = 10, *P* = 0.8507; C: *n* = 10, *P* = 0.1182; D: *n* = 6, *P* = 0.4693; two-tailed paired student’s t-test). Group data were presented as means ± S.E.M. n.s. no significance.


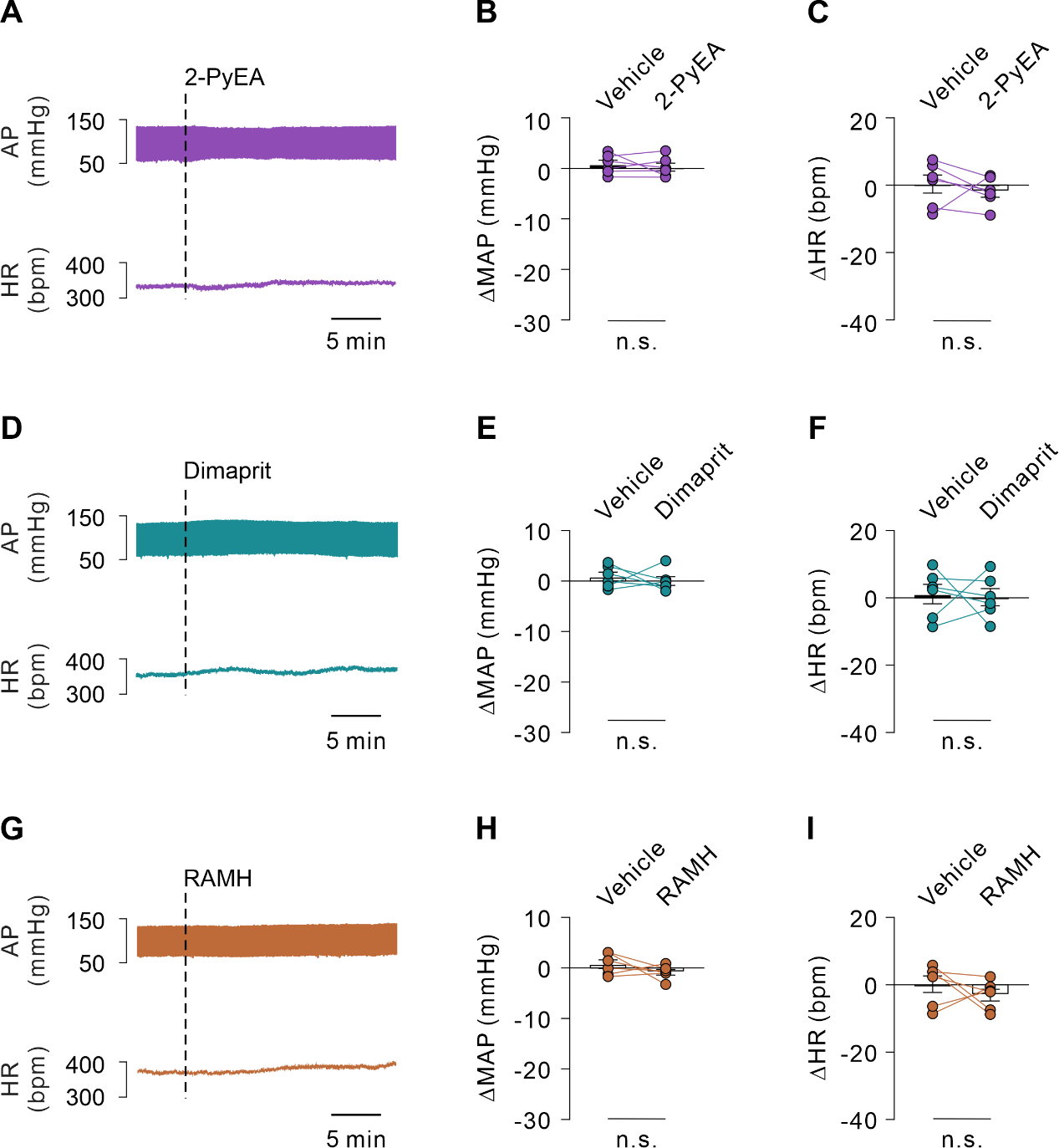


**Figure S3. Microinjection of selective H1R, H2R, and H3R agonists into RVMM does not influence MAP and HR in normal rats *in vivo*.** (**A**-**C**) Raw traces (A) and group data (B and C) for changes in AP and HR induced by microinjection of selective H1R agonist 2-PyEA into RVMM (B: *n* = 6, *P* = 0.5789; C: *n* = 6, *P* = 0.4812). (**D**-**F**) Raw traces (D) and group data (E and F) for changes in AP and HR induced by microinjection of selective H2R agonist dimaprit into RVMM (E: *n* = 6, *P* = 0.5894; F: *n* = 6, *P* = 0.8491). (**G**-**I**) Raw traces (G) and group data (H and I) for changes in AP and HR induced by microinjection of selective H3R agonist RAMH into RVMM (H: *n* = 6, *P* = 0.2494; I: *n* = 6, *P* = 0.4065). Group data were presented as means ± S.E.M and analyzed by paired student’s t-test. n.s. no significance.


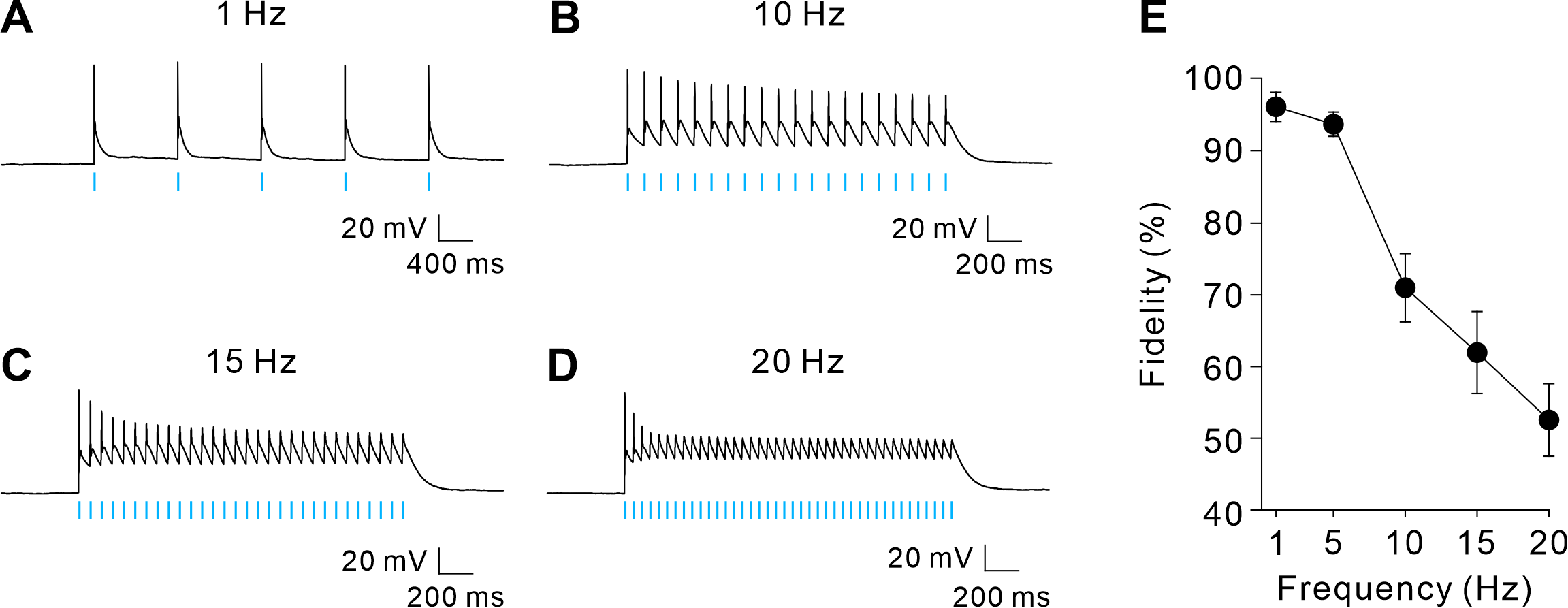


**Figure S4. Optogenetic stimulation of TMN histaminergic neurons transduced with ChR2 elicits action potentials.** (**A-D**) Representative traces showing action potential firing evoked by opto-stimulation at 1, 10, 15, and 20 Hz in ChR2-expressing TMN neurons. (**E**) Quantification of action potential fidelity (*n* = 11 for 1, 5, 10, and 20 Hz; *n* = 9 for 15 Hz). Data were presented as means ± S.E.M.


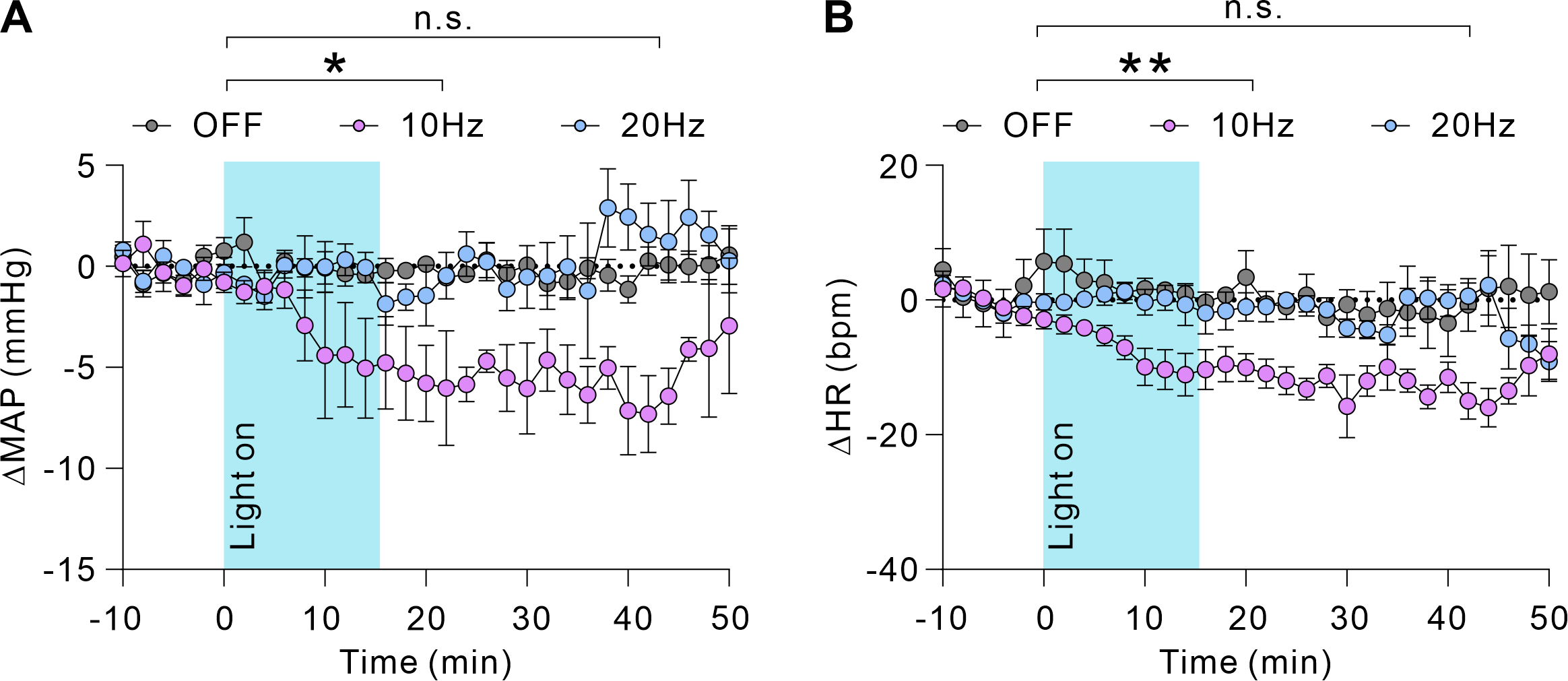


**Figure S5. Cardiovascular responses induced by optogenetic stimulation of TMN-RVMM histaminergic projections at different frequencies.** (**A**) Time-course of MAP changes during opto-stimulation at 10, 20 Hz and in the absence of light (OFF) (*n* = 5 per group; OFF *vs* 10 Hz: *P* = 0.0117, OFF *vs* 20 Hz: *P* = 0.8170). (**B**) Time-course of HR changes under the same conditions (*n* = 5 per group; OFF *vs* 10 Hz: *P* = 0.0014, OFF *vs* 20 Hz: *P* = 0.3215). Group data were presented as means ± S.E.M and analyzed by repeated measures two-way ANOVA with Bonferroni's multiple comparisons test. **P* < 0.05, ***P* < 0.01, n.s. no significance.


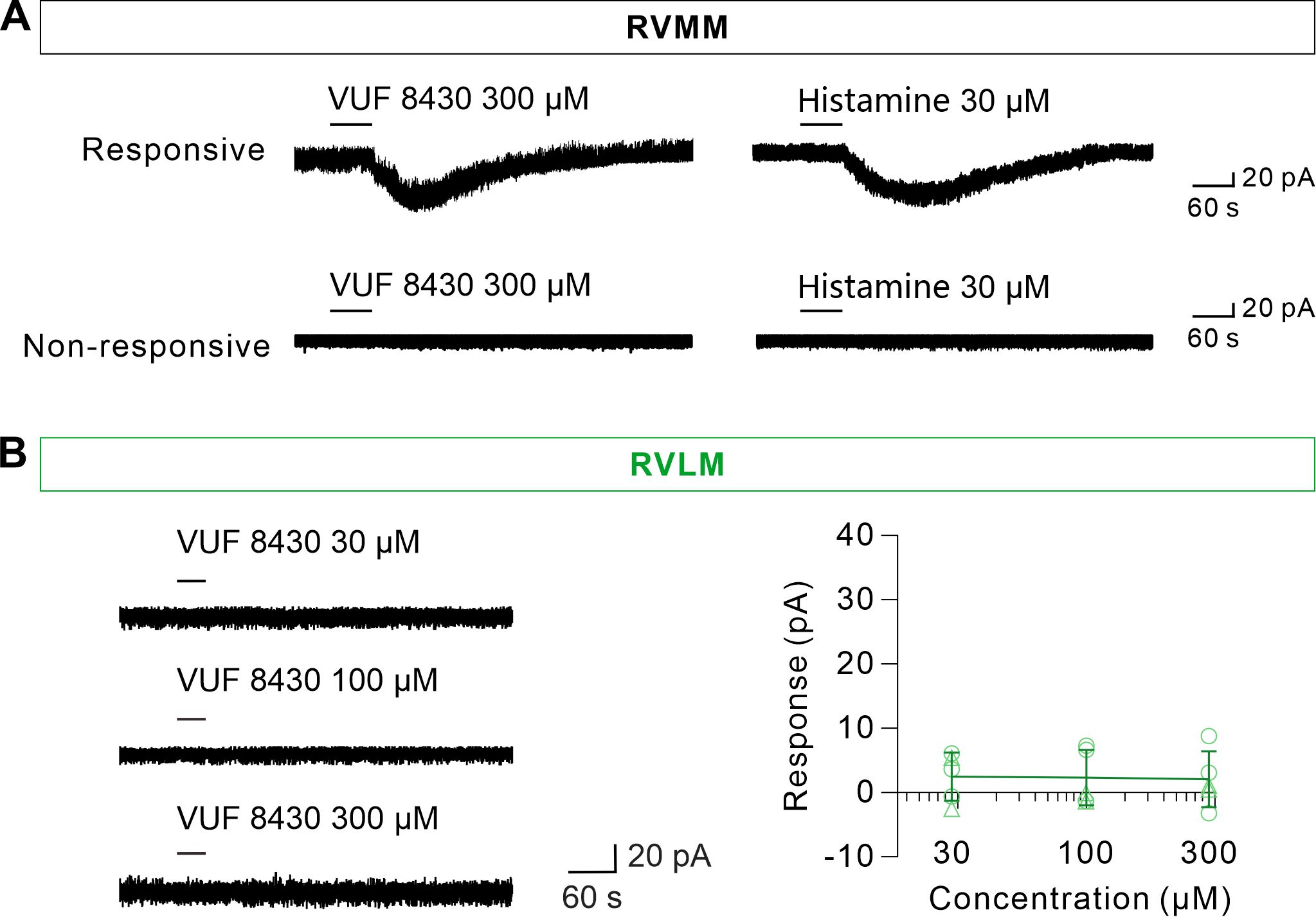


**Figure S6.** **Activation of H4R evokes inward currents on RVMM neurons but does not influence RVLM neurons.** (**A**) Representative raw traces respectively showing the responsive cells (*n* = 28) and non-responsive cells (*n* = 5) to VUF 8340 (300 μM) as well as the responsive cells (*n* = 30) and non-responsive cells (*n* = 14) to 30 μM histamine in RVMM. (**B**) Raw traces and group data showed that 30 μM, 100 μM, and 300 μM VUF 8430 did not affect RVLM neurons (*n* = 5, circles for 3 male rats and triangles for 2 female rats). Group data were presented as means ± S.E.M.


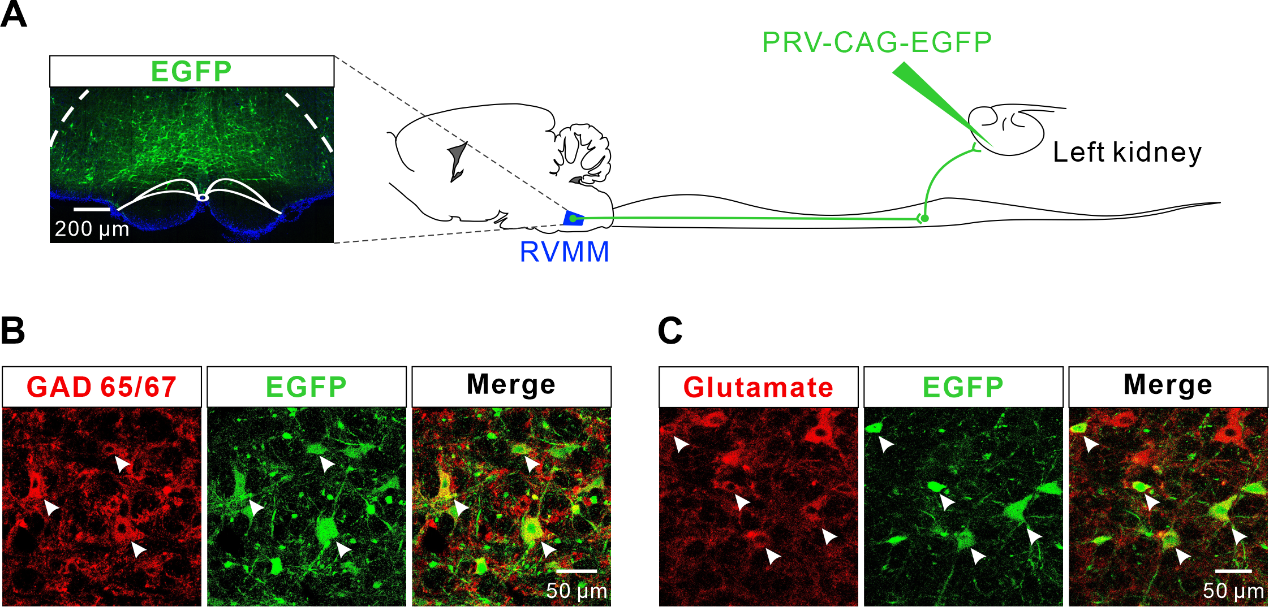


**Figure S7. Retrograde transneuronal tracing inhibitory and excitatory sympathetic efferent pathways from RVMM to the kidney.** (**A**) Microinjection of retrograde transneuronal tracing virus, PRV-CAG-EGFP, into the left kidney for tracing sympathetic efferent pathways derived from RVMM. Scale bar, 200 µm. (**B**) The subpopulation of inhibitory presympathetic neurons in RVMM was GAD 65/67 immunoreactive, and EGFP fluorescence co-localized. Scale bar, 50 µm. (**C**) The subpopulation of excitatory presympathetic neurons in RVMM was glutamate staining, and EGFP fluorescence co-localized. Scale bar, 50 µm.


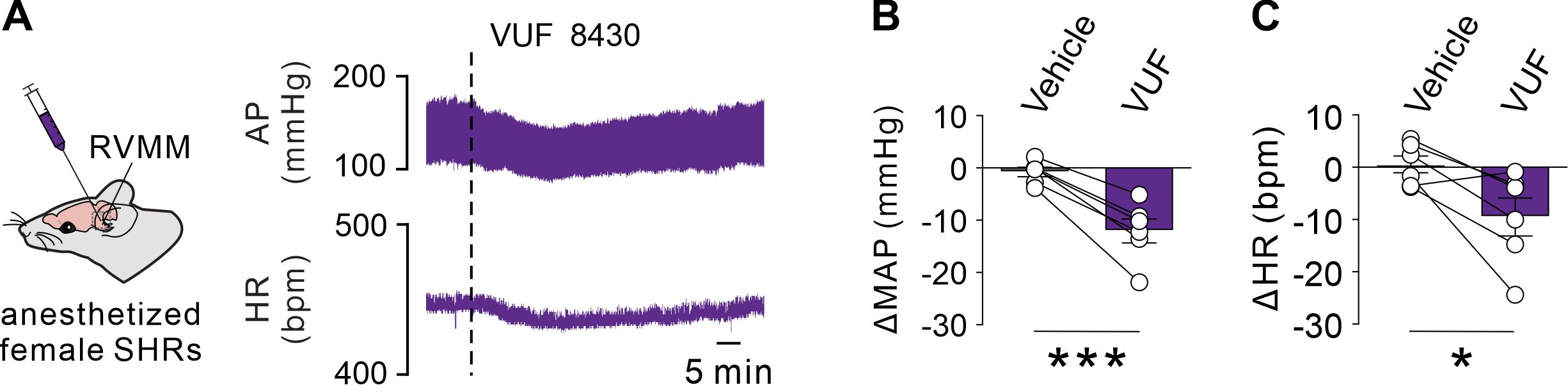


**Figure S8. Activation of H4R in RVMM ameliorates hypertension in anesthetized female SHRs.** (**A**) Raw traces illustrating changes in MAP and HR induced by microinjection of VUF 8430 into RVMM of female SHRs. (**B** and **C**) Group data of maximal responses of MAP (B) and HR (C) after microinjection of VUF 8430 or vehicle into RVMM of female SHRs (B: *n* = 6; *P* = 0.0009; C: *n* = 6, *P* = 0.0303; two-tailed paired student’s t-test). Group Data were presented as means ± S.E.M. **P* < 0.05, ****P* < 0.001.


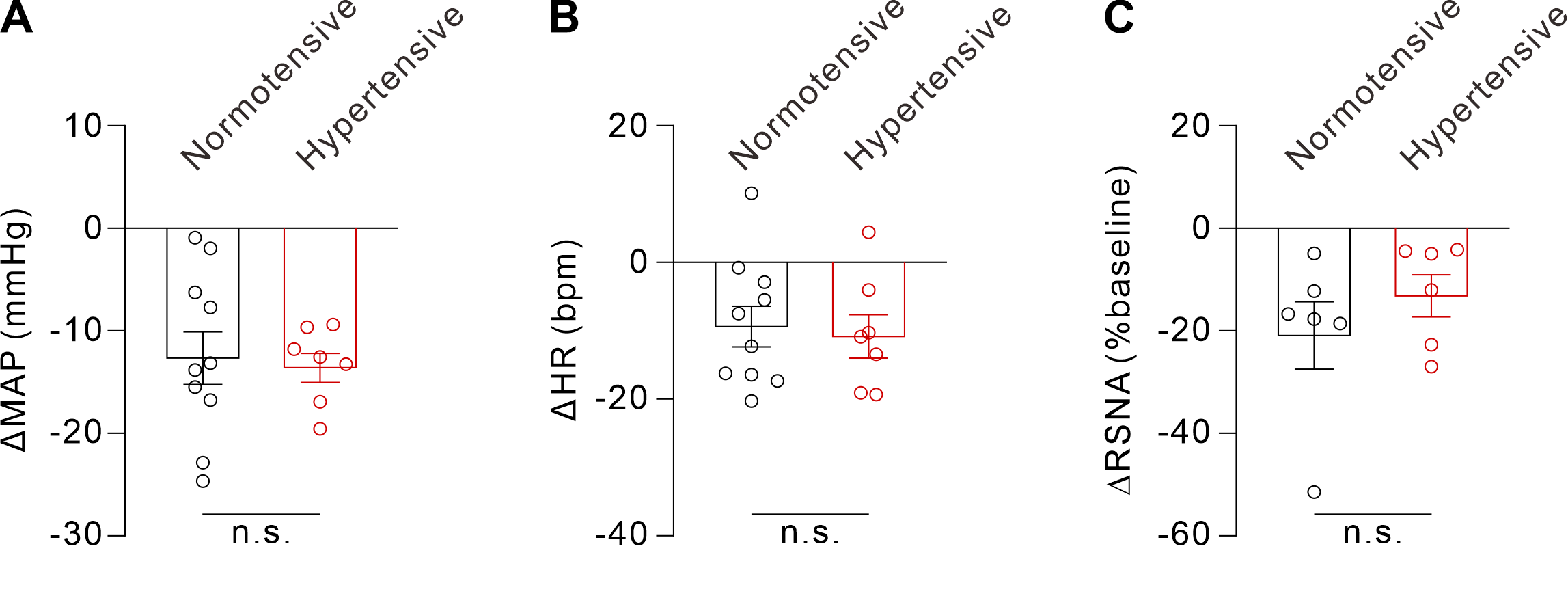


**Figure S9. Comparable net cardiovascular responses to H4R activation in the RVMM of normotensive and hypertensive rats.** (**A**) Group data showing net MAP responses following VUF 8430 microinjection into the RVMM (*n* = 10 for Normotensive, *n* = 7 for Hypertensive; *P* = 0.7789). (**B**) Group data showing net HR responses under the same conditions (*n* = 10 for Normotensive, *n* = 7 for Hypertensive; *P* = 0.7472). (**C**) Group data showing net RSNA responses under the same conditions (*n* = 10 for Normotensive, *n* = 7 for Hypertensive; *P* = 0.3422). Data were presented as means ± S.E.M and analyzed using two-tailed unpaired student’s t-test. n.s. no significance.


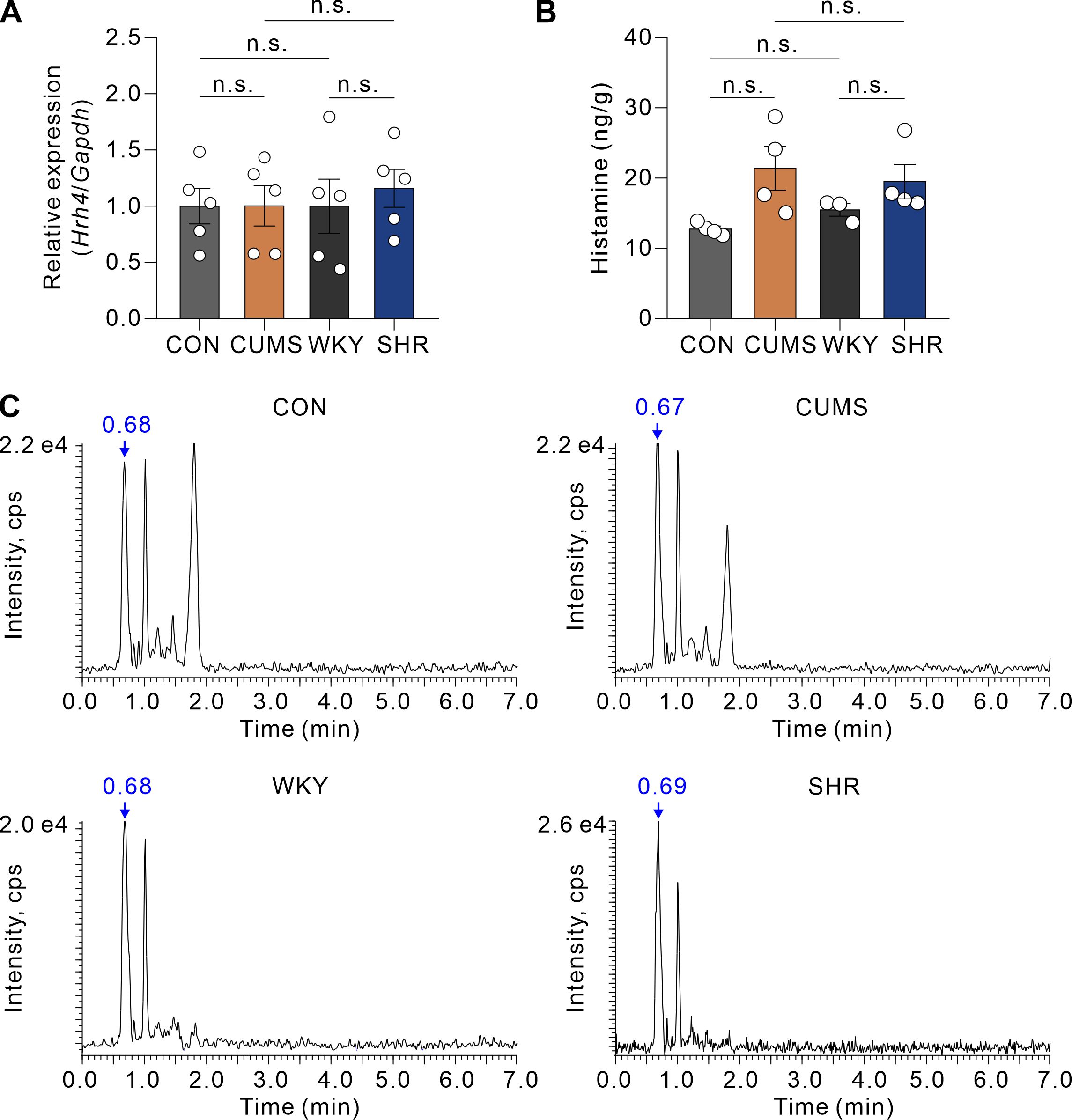


**Figure S10. Detection of H4R expression and histamine levels in RVMM of normotensive and hypertensive rats.** (**A**) Relative expression of *Hrh4* in CON (wildtype Sprague-Dawley rats without CUMS), CUMS-induced hypertensive rats, normotensive WKY and SHRs (*n* = 5 for per group; CON *vs* CUMS: *P* > 0.9999, CON *vs* WKY: *P* > 0.9999, WKY *vs* SHR: *P* > 0.9999, CUMS *vs* SHR: *P* > 0.9999; one-way ANOVA with Bonferroni's multiple comparisons test). (**B**) The histamine levels in RVMM (*n* = 4 or 3 for per group; CON *vs* CUMS: *P* = 0.0861, CON *vs* WKY: *P* > 0.9999, WKY *vs* SHR: *P* > 0.9999, CUMS *vs* SHR: *P* = 0.5583; one-way ANOVA with Bonferroni's multiple comparisons test). (**C**) Representative extraction chromatograms of multiple-reaction monitoring chromatograms of per group in (B). Arrows show the chromatographic peak of histamine. Group data were presented as means ± S.E.M. n.s. no significance.


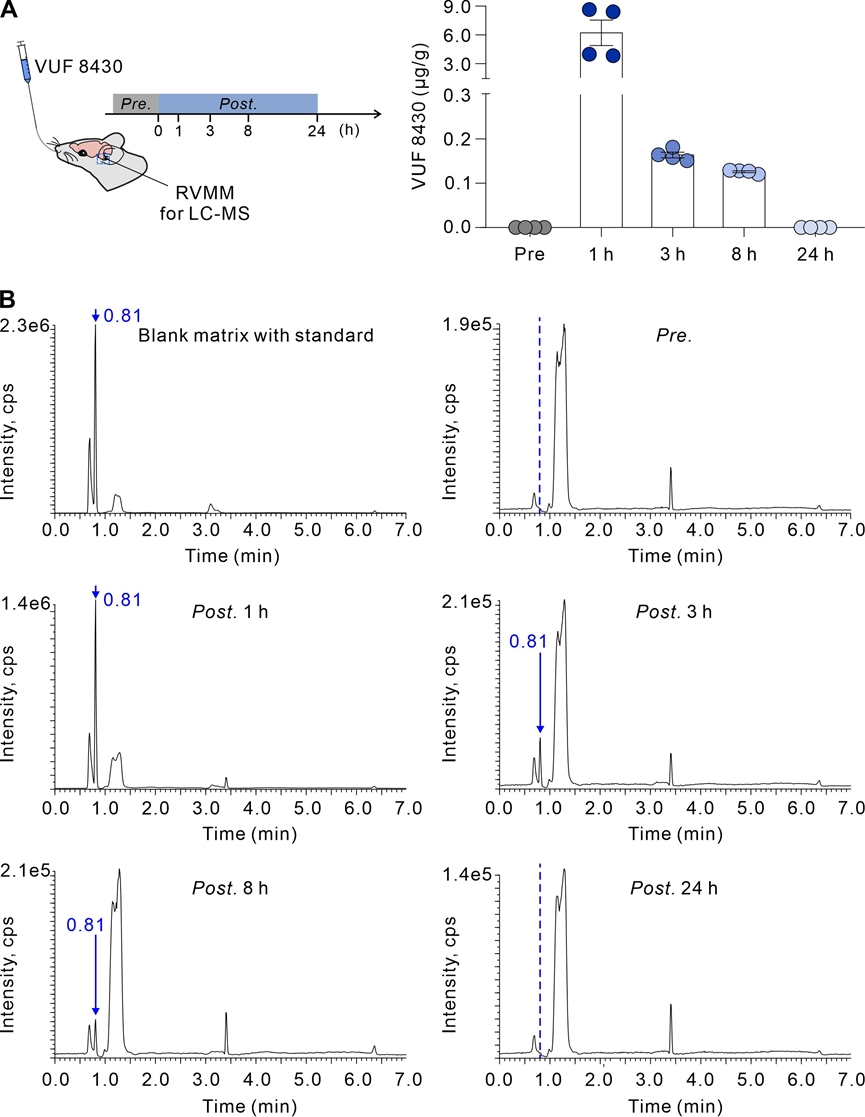


**Figure S11. Detection of VUF 8430 concentration in RVMM after intranasal delivery in SHRs.** (**A**) Group data of VUF concentration in RVMM by using high-performance liquid chromatography-mass spectrometry (LC-MS) analysis (*n* = 4 per group). (**B**) Representative extraction chromatograms of multiple-reaction monitoring chromatograms of blank matrix with VUF standard and RVMM tissue before intranasal administration and at 1, 3, 8, and 24 h after administration. Arrows show the chromatographic peak of VUF 8430. Dash lines indicate none of the chromatographic peaks of VUF 8430.


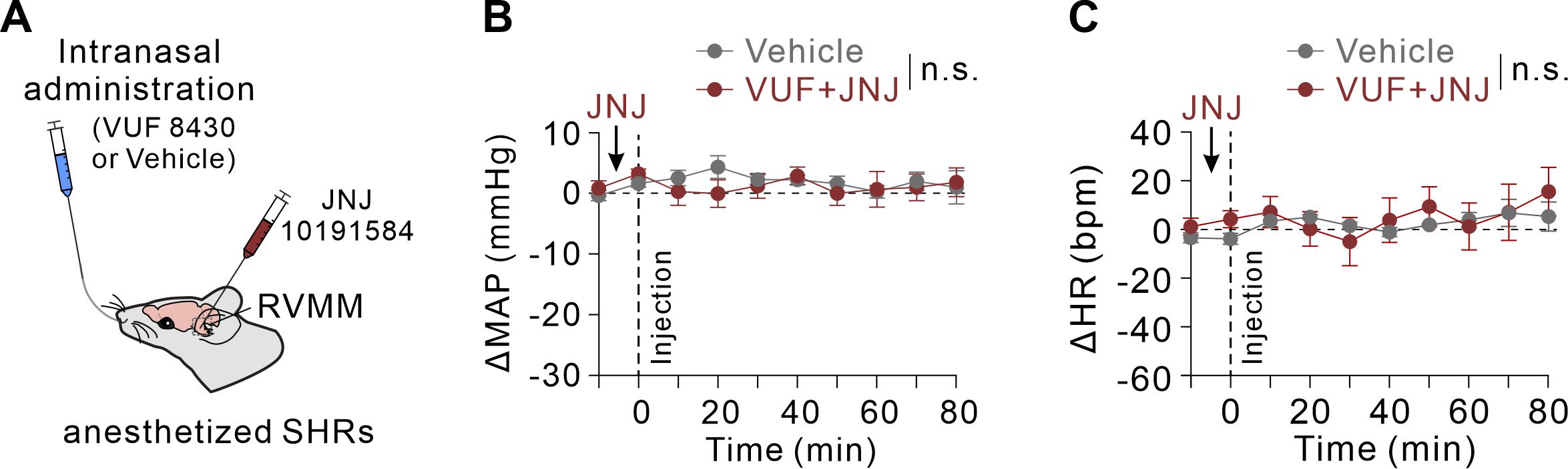


**Figure S12. Blockage of H4R in RVMM by H4R selective antagonist JNJ 10191584 abolished hypotensive effects induced by intranasal delivery of VUF 8430.** (**A**) Scheme of experimental blockage manipulation. Bilateral pre-microinjection of JNJ 10191584 (30 µM in a volume of 1 μL each side) into RVMM 5 min before intranasal administration of VUF or Vehicle. (**B**) Group data showed that pre-microinjection of H4R antagonist JNJ 10191584 into RVMM totally blocks depressor responses induced by intranasal administration of VUF (*n* = 5 for per group, *P* = 0.7481; repeated measures two-way ANOVA with Bonferroni's multiple comparisons test). (**C**) Group data showed that pre-microinjection of H4R antagonist JNJ 10191584 into RVMM totally blocks bradycardia responses induced by intranasal administration of VUF (*n* = 5 for per group, *P* = 0.7302; repeated measures two-way ANOVA with Bonferroni's multiple comparisons test). Group Data were presented as means ± S.E.M. n.s. no significance.


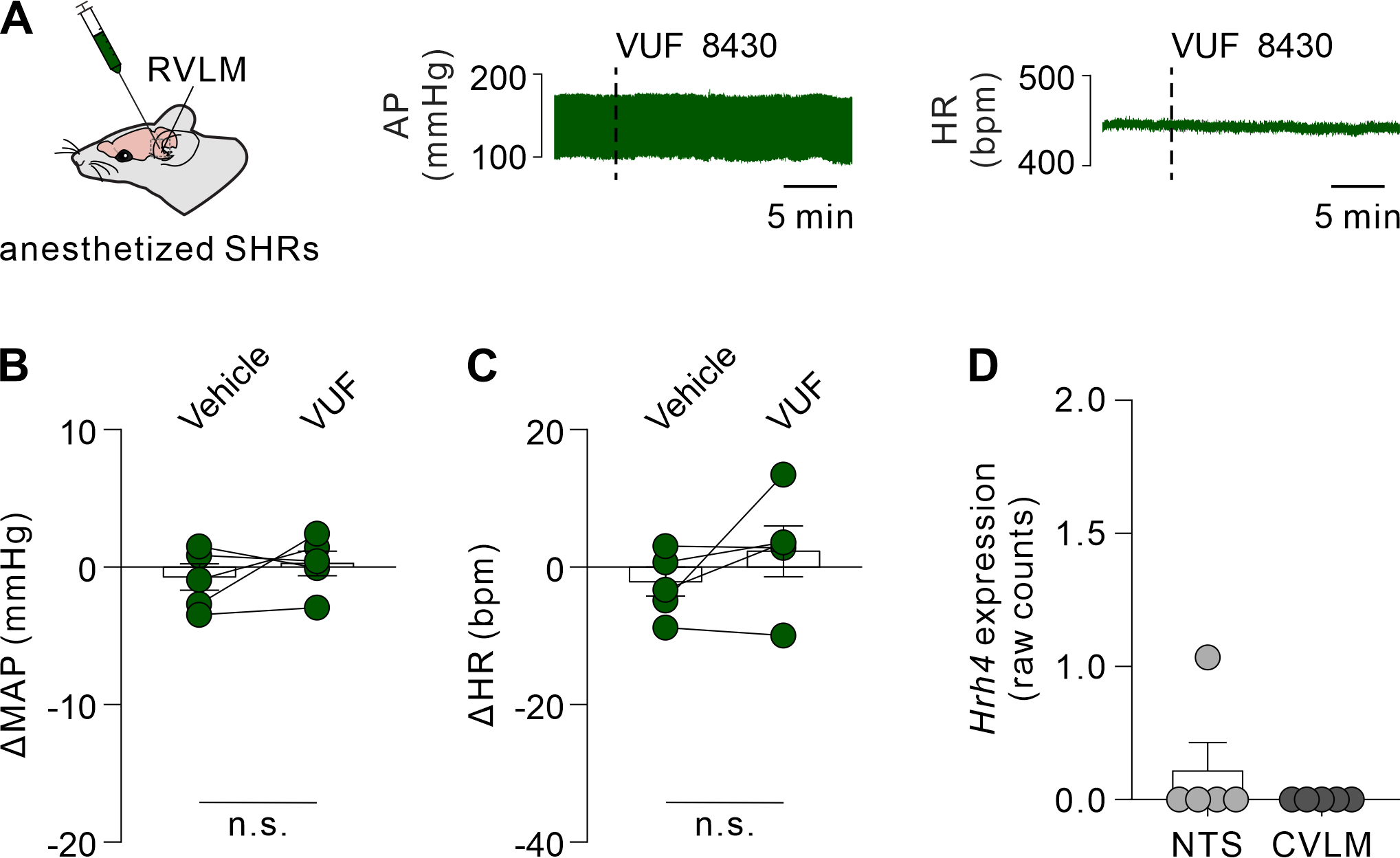


**Figure S13. RVLM, NTS or CVLM does not contribute to cardiovascular effects induced by intranasal activation of H4R.** (**A**) Raw traces illustrating changes in AP and HR induced by microinjection of VUF 8430 into RVLM. (**B** and **C**) Group data of maximal responses of MAP (B) and HR (C) after microinjection of VUF 8430 or vehicle into RVLM (B: *n* = 5, *P* = 0.3636; C: *n* = 5, *P* = 0.2062; two-tailed paired student’s t-test). (**D**) Expression of *Hrh4* in NTS and CVLM of rats based on publicly available sequencing data (GSE234784). In the NTS, only a single sample contained one read of *Hrh4*, indicating an exceedingly low expression level of H4R in this region. No *Hrh4* expression was detected in the CVLM (*n* =5 rats per group). Group data were presented as means ± S.E.M. n.s. no significance.


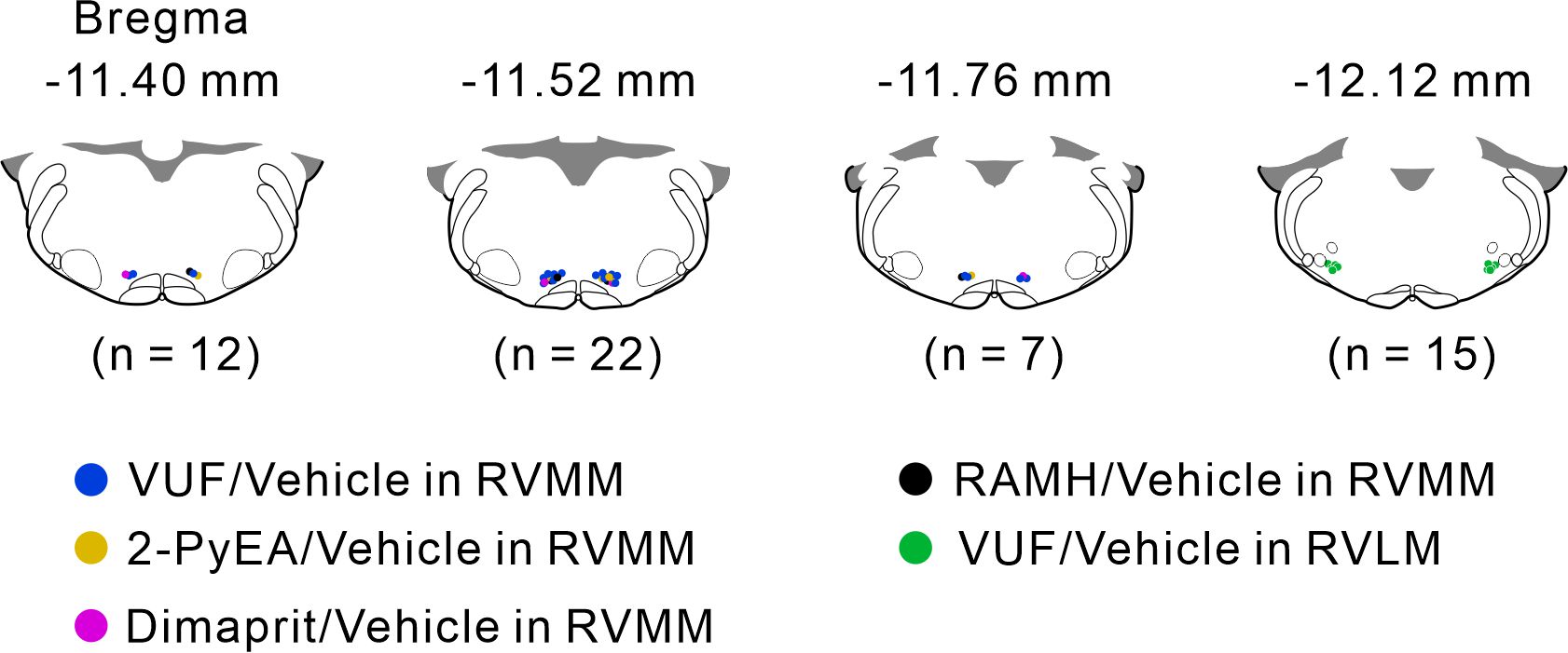


**Figure S14. Reconstruction of microinjection sites in RVMM and RVLM.** Distributions of microinjection sites in RVMM and RVLM in coronal planes for all groups are shown in Figure 1, Figure 7, and Figure S2, S3, S8, S9, and S13.

**Table S1. Baseline of MAP and HR in pharmacological and optogenetic manipulation of the anaesthetized rats.**

| **Figures** | **Groups** | ***n*** | **Before (mean ± SEM)** | | **After (mean ± SEM)** | | **Peak changes**  **(mean ± SEM)** | |
| --- | --- | --- | --- | --- | --- | --- | --- | --- |
|  |  |  | **MAP (mmHg)** | **HR**  **(bpm)** | **MAP (mmHg)** | **HR**  **(bpm)** | **MAP (mmHg)** | **HR**  **(bpm)** |
| **Fig. 1H-J** | Vehicle | 10 | 102.1 ± 2.36 | 383.5 ± 14.55 | 101.7 ± 2.41 | 380.4 ± 15.10 | -0.37 ± 0.37 | -3.11 ± 2.00 |
|  | VUF | 10 | 101.4 ± 0.94 | 379.1 ± 10.54 | 88.68 ± 2.62 | 367.1 ± 9.45 | -12.72 ± 2.32 | -12.01 ± 2.89 |
| **Fig. 2B, C** | Control | 6 | 97.49 ± 2.98 | 370.0 ± 8.71 | 99.25 ± 3.39 | 370.4 ± 10.48 | 1.76 ± 0.99 | 0.37 ± 2.08 |
|  | ChR2 | 6 | 96.79 ± 1.52 | 366.2 ± 8.25 | 91.65 ± 2.13 | 343.5 ± 5.67 | -5.13 ± 1.52 | -22.64 ± 5.93 |
| **Fig. 2D, E** | + Vehicle | 5 | 99.96 ± 1.73 | 372.7 ± 5.88 | 94.83 ± 1.28 | 350.2 ± 10.17 | -5.13 ± 0.78 | -22.53 ± 5.74 |
|  | + JNJ | 5 | 99.18 ± 0.94 | 372.2 ± 6.59 | 100.1 ± 2.27 | 380.1 ± 11.33 | 0.91 ± 1.43 | 7.93 ± 8.74 |
| **Fig. 5G, H** | + Vehicle | 5 | 97.48 ± 5.17 | 365.5 ± 7.50 | 91.53 ± 5.41 | 345.9 ± 7.56 | -5.95 ± 2.26 | -19.56 ± 6.03 |
|  | + AMG | 5 | 97.08 ± 5.90 | 351.8 ± 17.07 | 97.67 ± 6.54 | 356.4 ± 18.27 | 0.59 ± 1.25 | 4.57 ± 1.51 |
| **Fig. 7E-G** | Vehicle | 7 | 145.3 ± 4.89 | 421.8 ± 9.25 | 144.0 ± 4.86 | 421.0 ± 10.01 | -1.29 ± 0.63 | -0.83 ± 1.79 |
|  | VUF | 7 | 143.8 ± 4.96 | 419.7 ± 6.37 | 130.9 ± 3.97 | 410.8 ± 4.80 | -12.93 ± 1.93 | -8.92 ± 2.66 |
| **Fig. 7I, J** | WKY-Vehicle | 5 | 102.6 ± 4.65 | 387.0 ± 10.74 | 102.8 ± 2.97 | 385.1 ± 9.66 | 0.18 ± 2.09 | -1.93 ± 2.42 |
|  | WKY-VUF | 5 | 102.1 ± 3.69 | 383.1 ± 11.79 | 89.02 ±4.96 | 351.5 ± 19.77 | -13.10 ± 3.74 | -31.60 ± 10.04 |
|  | SHR-Vehicle | 5 | 140.2 ± 6.40 | 430.0 ± 13.10 | 139.4 ± 6.55 | 425.3 ± 13.43 | -0.84 ± 1.38 | -4.71 ± 9.73 |
|  | SHR-VUF | 5 | 141.0 ± 5.61 | 427.6 ± 11.96 | 129.2 ± 6.90 | 392.7 ± 16.74 | -11.76 ± 2.00 | -34.87 ± 9.86 |
| **Fig. S2B-D** | Vehicle | 10 | 98.14 ± 1.84 | 365.7 ± 9.87 | 98.10 ± 1.68 | 368.4 ± 10.00 | -0.04 ± 0.86 | 2.79 ± 1.90 |
|  | VUF | 10 | 98.72 ± 1.81 | 372.5 ± 10.37 | 99.00 ± 2.38 | 370.8 ± 10.92 | 0.29 ± 1.03 | -1.62 ± 2.14 |
| **Fig. S3B, C** | Vehicle | 6 | 97.64 ± 4.93 | 367.3 ± 12.86 | 98.47 ± 5.56 | 367.7 ± 13.76 | 0.82 ± 0.78 | 0.36 ± 2.70 |
|  | 2-PyEA | 6 | 98.49 ± 4.37 | 364.8 ± 16.31 | 98.76 ± 4.92 | 363.0 ± 15.42 | 0.26 ± 0.80 | -1.82 ± 1.75 |
| **Fig. S3E, F** | Vehicle | 6 | 99.79 ± 4.05 | 373.4 ± 11.77 | 100.7 ± 4.27 | 374.6 ± 12.99 | 0.89 ± 0.88 | 1.14 ± 2.87 |
|  | Dimaprit | 6 | 96.36 ± 3.06 | 369.2 ± 7.03 | 96.38 ± 3.56 | 369.4± 7.10 | 0.02 ± 0.86 | 0.23 ± 2.56 |
| **Fig. S3H, I** | Vehicle | 6 | 101.4 ± 2.84 | 381.2 ± 5.25 | 102.2 ± 2.59 | 381.4 ± 4.34 | 0.77 ± 0.86 | 0.18 ± 2.48 |
|  | RAMH | 6 | 101.5 ± 2.65 | 360.7 ± 9.64 | 100.7 ± 2.63 | 357.7 ± 10.96 | -0.84 ± 0.56 | -3.04 ± 1.74 |
| **Fig. S5A, B** | OFF | 5 | 97.66 ± 1.53 | 370.8 ± 8.33 | 97.57 ± 1.29 | 370.2 ± 9.98 | -0.10 ± 0.52 | -0.65 ± 2.20 |
|  | 10 Hz | 5 | 99.40 ± 2.80 | 366.0 ± 9.45 | 93.03 ± 3.52 | 350.2 ± 11.56 | -6.36 ± 1.40 | -15.77 ± 4.67 |
|  | 20 Hz | 5 | 96.73 ± 1.87 | 361.7 ± 8.53 | 95.52 ± 4.12 | 357.6 ± 8.43 | -1.21 ± 3.35 | -4.16 ± 1.38 |
| **Fig. S8B, C** | Vehicle | 6 | 141.5 ± 3.12 | 424.3 ± 10.27 | 140.7 ± 3.08 | 424.8 ± 11.14 | -0.80 ± 0.88 | 0.52 ± 1.64 |
|  | VUF | 6 | 141.5 ± 3.52 | 426.6 ± 9.29 | 129.4 ± 3.69 | 417.1 ± 10.25 | -12.06 ± 2.30 | -9.52 ± 3.63 |
| **Fig. S12B, C** | Vehicle | 5 | 140.2 ± 2.01 | 420.4 ± 10.55 | 140.5 ± 1.55 | 424.3 ± 11.96 | 0.26 ± 1.05 | 4.00 ± 3.10 |
|  | VUF + JNJ | 5 | 139.7 ± 2.47 | 418.3 ± 10.66 | 140.4 ± 2.39 | 419.6 ± 17.95 | 0.65 ± 2.93 | 1.33 ± 9.68 |
| **Fig. S13B, C** | Vehicle | 5 | 140.4 ± 1.72 | 429.8 ± 5.28 | 139.5 ± 2.26 | 427.3 ± 4.65 | -0.94 ± 0.96 | -2.57 ± 2.08 |
|  | VUF | 5 | 139.4 ± 2.61 | 422.0 ± 6.29 | 139.6 ± 3.18 | 424.8 ± 6.18 | 0.26 ± 0.91 | 2.75 ± 3.72 |

**Table S2. Statistical analysis.**

| **Figures** | ***n*** | **Test used** | **Statistical results** |
| --- | --- | --- | --- |
| **Fig. 1E** | n = 5 rats |  |  |
| **Fig. 1F** | RVMM, n = 5 rats  RVLM, n = 5 rats | Two-tailed unpaired t test | t (8) = 0.2246, *P* = 0.8279 |
| **Fig. 1H** | Vehicle, n = 10 rats  VUF, n = 10 rats | Two-tailed paired t test | t (9) = 4.823, *P* = 0.0009 |
| **Fig. 1I** | Vehicle, n = 10 rats  VUF, n = 10 rats | Two-tailed paired t test | t (9) = 2.987, *P* = 0.0153 |
| **Fig. 1J** | Vehicle, n = 6 rats  VUF, n = 6 rats | Two-tailed paired t test | t (5) = 3.089, *P* = 0.0272 |
| **Fig. 2B** | Control, n = 6 rats  ChR2, n = 6 rats | RM two-way ANOVA  *Bonferroni's multiple comparisons test* | group: F (1, 10) = 14.76; Time: F (30, 300) = 1.956; Interaction: F (30, 300) = 4.230;  Control *vs* ChR2: *P* = 0.0033 |
| **Fig. 2C** | Control, n = 6 rats  ChR2, n = 6 rats | RM two-way ANOVA  *Bonferroni's multiple comparisons test* | group: F (1, 10) = 12.90; Time: F (30, 300) = 2.257; Interaction: F (30, 300) = 2.286;  Control *vs* ChR2: *P* = 0.0049 |
| **Fig. 2D** | ChR2 + JNJ 10191584, n = 5 rats  ChR2 + Vehicle, n = 5 rats | RM two-way ANOVA  *Bonferroni's multiple comparisons test* | group: F (1, 8) = 16.68; Time: F (35, 280) = 1.790; Interaction: F (35, 280) = 3.261;  ChR2 + JNJ 10191584 *vs* ChR2 + Vehicle: *P* = 0.0035 |
| **Fig. 2E** | ChR2 + JNJ 10191584, n = 5 rats  ChR2 + Vehicle, n = 5 rats | RM two-way ANOVA  *Bonferroni's multiple comparisons test* | group: F (1, 8) = 8.215; Time: F (35, 280) = 1.477; Interaction: F (35, 280) = 3.517;  ChR2 + JNJ 10191584 *vs* ChR2 + Vehicle: *P* = 0.0210 |
| **Fig. 3B** | 6 cells from 6 rats |  |  |
| **Fig. 3D** | WT, n = 5 mouse  KO, n = 5 mouse | Two-tailed unpaired t test | t (8) = 8.786, *P* < 0.0001 |
| **Fig. 3F** | WT, n = 6 cells from 6 mouse;  KO, n = 5 cells for 5 mouse |  |  |
| **Fig. 4B** | 4 cells from 4 rats |  |  |
| **Fig. 4D** | Histamine, n = 6 cells from 6 rats  + JNJ 3 μM, n = 6 cells from 6 rats  + JNJ 10 μM, n = 6 cells from 6 rats  + JNJ 30 μM, n = 6 cells from 6 rats | RM one-way ANOVA  *Bonferroni's multiple comparisons test* | F (2.108, 10.52) = 162.2;  Histamine *vs* + JNJ 3 μM: *P* = 0.0005;  Histamine *vs* + JNJ 10 μM: *P* < 0.0001;  Histamine *vs* + JNJ 30 μM: *P* < 0.0001 |
| **Fig. 4F** | VUF, n = 5 cells from 5 rats  + JNJ 3 μM, n = 5 cells from 5 rats  + JNJ 10 μM, n = 5 cells from 5 rats  + JNJ 30 μM, n = 5 cells from 5 rats | RM one-way ANOVA  *Bonferroni's multiple comparisons test* | F (2.002, 8.009) = 80.13;  VUF *vs* + JNJ 3 μM: *P* = 0.0006;  VUF *vs* + JNJ 10 μM: *P* = 0.0010;  VUF *vs* + JNJ 30 μM: *P* = 0.0004 |
| **Fig. 4H** | 7 cells from 7 rats |  |  |
| **Fig. 4J** | VUF, n = 5 cells from rats  + TTX + NBQX + AP5 + SR 95531, n = 5 cells from 5 rats | Two-tailed paired t test | t (4) = 1.377, *P* = 0.2406 |
| **Fig. 5B** | Histamine, n = 8 cells from 8 rats  + AMG 9810, n = 8 cells from 8 rats | Two-tailed paired t test | t (7) = 19.36, *P* < 0.0001 |
| **Fig. 5C** | VUF, n = 6 cells from 6 rats  + AMG 9810, n = 6 cells from 6 rats | Two-tailed paired t test | t (5) = 17.08, *P* < 0.0001 |
| **Fig. 5E** | RVMM, n = 4 rats  RVLM, n = 4 rats | Two-tailed unpaired t test | t (6) = 2563, *P* < 0.0001 |
| **Fig. 5G** | ChR2 + AMG 9810, n = 5 rats  ChR2 + Vehicle, n = 5 rats | RM two-way ANOVA  *Bonferroni's multiple comparisons test* | group: F (1, 8) = 7.753; Time: F (35, 280) = 2.619; Interaction: F (35, 280) = 3.163;  ChR2 + AMG 9810 *vs* ChR2 + Vehicle: *P* = 0.0238 |
| **Fig. 5H** | ChR2 + AMG 9810, n = 5 rats  ChR2 + Vehicle, n = 5 rats | RM two-way ANOVA  *Bonferroni's multiple comparisons test* | group: F (1, 8) = 7.268; Time: F (35, 280) = 0.4830; Interaction: F (35, 280) = 3.731;  ChR2 + AMG 9810 *vs* ChR2 + Vehicle: *P* = 0.0272 |
| **Fig. 6G** | GAD, n = 5 rats  Glutamate, n = 5 rats | Two-tailed unpaired t test | t (8) = 6.866, *P* = 0.0001 |
| **Fig. 7E** | Vehicle, n = 7 rats  VUF, n = 7 rats | Two-tailed paired t test | t (6) = 8.226, *P* = 0.0002 |
| **Fig. 7F** | Vehicle, n = 7 rats  VUF, n = 7 rats | Two-tailed paired t test | t (6) = 3.385, *P* = 0.0148 |
| **Fig. 7G** | Vehicle, n = 6 rats  VUF, n = 6 rats | Two-tailed paired t test | t (5) = 3.053, *P* = 0.0283 |
| **Fig. 7I** | WKY-Vehicle, n = 5 rats;  WKY-VUF, n = 5 rats;  SHR-Vehicle, n = 5 rats;  SHR-VUF, n = 5 rats | RM two-way ANOVA  *Bonferroni's multiple comparisons test* | Interaction: F (27, 144) = 5.871, *P* < 0.0001;  WKY-Vehicle *vs* WKY-VUF: *P* = 0.0034; group: F (1, 8) = 16.80; Time: F (9, 72) = 4.252; Interaction: F (9, 72) = 7.250;  SHR-Vehicle *vs* SHR-VUF: *P* = 0.0086; group: F (1, 8) = 11.98; Time: F (9, 72) = 10.37; Interaction: F (9, 72) = 9.690;  WKY-VUF *vs* SHR-VUF: *P* = 0.6344; group: F (1, 8) = 0.2442; Time: F (9, 72) = 23.39; Interaction: F (9, 72) = 1.896 |
| **Fig. 7J** | WKY-Vehicle, n = 5 rats;  WKY-VUF, n = 5 rats;  SHR-Vehicle, n = 5 rats;  SHR-VUF, n = 5 rats | RM two-way ANOVA  *Bonferroni's multiple comparisons test* | Interaction: F (27, 144) = 3.296, *P* < 0.0001;  WKY-Vehicle *vs* WKY-VUF: *P* = 0.0140; group: F (1, 8) = 9.793; Time: F (9, 72) = 5.232; Interaction: F (9, 72) = 4.691;  SHR-Vehicle vs SHR-VUF: *P* = 0.0315; group: F (1, 8) = 6.772; Time: F (9, 72) = 5.344; Interaction: F F (9, 72) = 4.959;  WKY-VUF *vs* SHR-VUF: *P* = 0.7359; group: F (1, 8) = 0.1219; Time: F (9, 72) = 15.17; Interaction: F (9, 72) = 0.3086 |
| **Fig. 8C** | Vehicle, n = 7 rats  VUF, n = 7 rats | RM two-way ANOVA  *Bonferroni's multiple comparisons test* | group: F (1, 12) = 6.255; Time: F (3, 36) = 6.787; Interaction: F (3, 36) = 6.926;  Vehicle *vs* VUF: *P* = 0.0279 |
| **Fig. 8D** | Vehicle, n = 7 rats  VUF, n = 7 rats | RM two-way ANOVA  *Bonferroni's multiple comparisons test* | group: F (1, 12) = 7.767; Time: F (3, 36) = 4.460; Interaction: F (3, 36) = 6.747;  Vehicle *vs* VUF: *P* = 0.0164 |
| **Fig. 8E** | Vehicle, n = 7 rats  VUF, n = 7 rats | RM two-way ANOVA  *Bonferroni's multiple comparisons test* | group: F (1, 12) = 6.281; Time: F (3, 36) = 4.250; Interaction: F (3, 36) = 7.013;  Vehicle *vs* VUF: *P* = 0.0276 |
| **Fig. 8F** | Vehicle, n = 7 rats  VUF, n = 7 rats | RM two-way ANOVA  *Bonferroni's multiple comparisons test* | group: F (1, 12) = 13.50; Time: F (3, 36) = 5.980; Interaction: F (3, 36) = 3.986;  Vehicle *vs* VUF: *P* = 0.0032 |
| **Fig. 8H** | Vehicle, n = 5 rats  VUF, n = 5 rats | mixed-effects analysis with *Bonferroni's multiple comparisons test* | group: F (1, 8) = 22.22; Time: F (5, 38) = 5.672;  Interaction: F (5, 38) = 4.873;  Vehicle *vs* VUF: *P* = 0.0015 |
| **Fig. 8I** | Vehicle, n = 5 rats  VUF, n = 5 rats | mixed-effects analysis with *Bonferroni's multiple comparisons test* | group: F (1, 8) = 12.84; Time: F (5, 38) = 5.349;  Interaction: F (5, 38) = 4.905;  Vehicle *vs* VUF: *P* = 0.0072 |
| **Fig. 8J** | Vehicle, n = 5 rats  VUF, n = 5 rats | mixed-effects analysis with *Bonferroni's multiple comparisons test* | group: F (1, 8) = 20.17; Time: F (5, 38) = 2.726;  Interaction: F (5, 38) = 2.959;  Vehicle *vs* VUF: *P* = 0.0020 |
| **Fig. 8L** | Control, n = 8 rats  CUMS, n = 10 rats | RM two-way ANOVA  *Bonferroni's multiple comparisons test* | group: F (1, 16) = 43.04; Time: F (7, 112) = 4.210; Interaction: F (7, 112) = 3.866;  Vehicle *vs* VUF: *P* < 0.0001 |
| **Fig. 8M** | Control, n = 8 rats  CUMS, n = 10 rats | RM two-way ANOVA  *Bonferroni's multiple comparisons test* | group: F (1, 16) = 26.69; Time: F (7, 112) = 3.542; Interaction: F (7, 112) = 3.654;  Vehicle *vs* VUF: *P* < 0.0001 |
| **Fig. 8O** | Vehicle, n = 5 rats  VUF, n = 5 rats | RM two-way ANOVA  *Bonferroni's multiple comparisons test* | group: F (1, 8) = 18.33; Time: F (5, 40) = 9.502; Interaction: F (5, 40) = 12.90;  Vehicle *vs* VUF: *P* = 0.0027 |
| **Fig. 8P** | Vehicle, n = 5 rats  VUF, n = 5 rats | RM two-way ANOVA  *Bonferroni's multiple comparisons test* | group: F (1, 8) = 95.77; Time: F (5, 40) = 6.494; Interaction: F (5, 40) = 9.809;  Vehicle *vs* VUF: *P* < 0.0001 |
| **Fig. 8Q** | Vehicle, n = 5 rats  VUF, n = 5 rats | RM two-way ANOVA  *Bonferroni's multiple comparisons test* | group: F (1, 8) = 36.45; Time: F (5, 40) = 7.105; Interaction: F (5, 40) = 7.544;  Vehicle *vs* VUF: *P* = 0.0003 |
| **Fig. S1B** | n = 3 rats |  |  |
| **Fig. S2B** | Vehicle, n = 10 rats  VUF, n = 10 rats | Two-tailed paired t test | t (9) = 0.1937, *P* = 0.8507 |
| **Fig. S2C** | Vehicle, n = 10 rats  VUF, n = 10 rats | Two-tailed paired t test | t (9) = 1.727, *P* = 0.1182 |
| **Fig. S2D** | Vehicle, n = 6 rats  VUF, n = 6 rats | Two-tailed paired t test | t (5) = 0.7825, *P* = 0.4693 |
| **Fig. S3B** | Vehicle, n = 6 rats  2-PyEA, n = 6 rats | Two-tailed paired t test | t (5) = 0.5932, *P* = 0.5789 |
| **Fig. S3C** | Vehicle, n = 6 rats  2-PyEA, n = 6 rats | Two-tailed paired t test | t (5) = 0.7607, *P* = 0.4812 |
| **Fig. S3E** | Vehicle, n = 6 rats  Dimaprit, n = 6 rats | Two-tailed paired t test | t (5) = 0.5763, *P* = 0.5894 |
| **Fig. S3F** | Vehicle, n = 6 rats  Dimaprit, n = 6 rats | Two-tailed paired t test | t (5) = 0.2004, *P* = 0.8491 |
| **Fig. S3H** | Vehicle, n = 6 rats  RAMH, n = 6 rats | Two-tailed paired t test | t (5) = 1.303, *P* = 0.2494 |
| **Fig. S3I** | Vehicle, n = 6 rats  RAMH, n = 6 rats | Two-tailed paired t test | t (5) = 0.9059, *P* = 0.4065 |
| **Fig. S4E** | 1 Hz, n = 11 cells from 5 rats  5 Hz, n = 11 cells from 5 rats  10 Hz, n = 11 cells from 5 rats  15 Hz, n = 9 cells from 5 rats  20 Hz, n = 11 cells from 5 rats |  |  |
| **Fig. S5A** | OFF, n = 5 rats  10 Hz, n = 5 rats  20 Hz, n = 5 rats | RM two-way ANOVA  *Bonferroni's multiple comparisons test* | Interaction: F (60, 360) = 2.017, *P* < 0.0001;  OFF *vs* 10 Hz: *P=*0.0117; group: F (1, 8) = 10.58; Time: F (30, 240) = 2.534; Interaction: F (30, 240) = 2.114;  OFF *vs* 20 Hz: *P=*0.8170; group: F (1, 8) = 0.0572; Time: F (30, 240) = 0.9856; Interaction: F (30, 240) = 1.058; |
| **Fig. S5B** | OFF, n = 5 rats  10 Hz, n = 5 rats  20 Hz, n = 5 rats | RM two-way ANOVA  *Bonferroni's multiple comparisons test* | Interaction: F (60, 360) = 1.417, *P* = 0.0296;  OFF *vs* 10 Hz: *P=*0.0014; group: F (1, 8) = 22.95; Time: F (30, 240) = 2.547; Interaction: F (30, 240) = 1.310;  OFF *vs* 20 Hz: *P=*0.3215; group: F (1, 8) = 1.117; Time: F (30, 240) = 0.8832; Interaction: F (30, 240) = 0.6254 |
| **Fig. S6B** | n = 5 cells from 5 rats |  |  |
| **Fig. S8B** | Vehicle, n = 6 rats  VUF, n = 6 rats | Two-tailed paired t test | t (5) = 7.057, *P* = 0.0009 |
| **Fig. S8C** | Vehicle, n = 6 rats  VUF, n = 6 rats | Two-tailed paired t test | t (5) = 2.994, *P* = 0.0303 |
| **Fig. S9A** | Normotensive, n = 10  Hypertensive, n = 7 | Two-tailed unpaired t test | t (15) = 0.2859, *P* = 0.7789 |
| **Fig. S9B** | Normotensive, n = 10  Hypertensive, n = 7 | Two-tailed unpaired t test | t (15) = 0.3284, *P* = 0.7472 |
| **Fig. S9C** | Normotensive, n = 10  Hypertensive, n = 7 | Two-tailed unpaired t test | t (10) = 0.9972, *P* = 0.3422 |
| **Fig. S10A** | CON, n = 5 rats;  CUMS, n = 5 rats;  WKY, n = 5 rats;  SHR, n= 5 rats | RM one-way ANOVA  *Bonferroni's multiple comparisons test* | F (3, 16) = 0.1726;  CON *vs* CUMS: *P* >0.9999;  CON *vs* WKY: *P* >0.9999;  WKY *vs* SHR: *P* >0.9999;  CUMS *vs* SHR: *P* >0.9999 |
| **Fig. S10B** | CON, n = 4 rats;  CUMS, n = 4 rats;  WKY, n = 3 rats;  SHR, n= 4 rats | RM one-way ANOVA  *Bonferroni's multiple comparisons test* | F (3, 11) = 3.368;  CON *vs* CUMS: *P* = 0.0861;  CON *vs* WKY: *P* >0.9999;  WKY *vs* SHR: *P* >0.9999;  CUMS *vs* SHR: *P* = 0.5583 |
| **Fig. S11A** | Pre, n = 4 rats;  1 h, n = 4 rats;  3 h, n = 4 rats;  8 h, n = 4 rats;  24 h, n = 4 rats |  |  |
| **Fig. S12B** | Vehicle, n = 5 rats;  VUF + JNJ, n = 5 rats | RM two-way ANOVA  *Bonferroni's multiple comparisons test* | Vehicle *vs* VUF + JNJ: *P* = 0.7481; group: F (1, 8) = 0.1105; Time: F (9, 72) = 0.6696; Interaction: F (9, 72) = 0.9207 |
| **Fig. S12C** | Vehicle, n = 5 rats;  VUF + JNJ, n = 5 rats | RM two-way ANOVA  *Bonferroni's multiple comparisons test* | Vehicle *vs* VUF + JNJ: *P* = 0.7302; group: F (1, 8) = 0.1276; Time: F (9, 72) = 1.774; Interaction: F (9, 72) = 0.9913 |
| **Fig. S13B** | Vehicle, n = 5 rats  VUF, n = 5 rats | Two-tailed paired t test | t (4) = 1.024, *P* = 0.3636 |
| **Fig. S13C** | Vehicle, n = 5 rats  VUF, n = 5 rats | Two-tailed paired t test | t (4) = 1.507, *P* = 0.2062 |
| **Fig. S13D** | NTS, n = 5 rats  CVLM, n = 5 rats |  |  |
